# Azide-functionalized ligand enabling organic–inorganic hybrid dielectric for high-performance solution-processed oxide transistors

**DOI:** 10.1038/s41467-022-34772-x

**Published:** 2022-11-17

**Authors:** Juhyeok Lee, Syed Zahid Hassan, Sangjun Lee, Hye Ryun Sim, Dae Sung Chung

**Affiliations:** Department of Chemical Engineering, Pohang University of Science and Technology (POSTECH), Pohang, 37673 Republic of Korea

**Keywords:** Electronic devices, Inorganic chemistry, Electronic materials, Organic chemistry, Metal-organic frameworks

## Abstract

We propose a highly efficient crosslinking strategy for organic–inorganic hybrid dielectric layers using azide-functionalized acetylacetonate, which covalently connect inorganic particles to polymers, enabling highly efficient inter- and intra-crosslinking of organic and inorganic inclusions, resulting in a dense and defect-free thin-film morphology. From the optimized processing conditions, we obtained an excellent dielectric strength of over 4.0 MV cm^−1^, a high dielectric constant of ~14, and a low surface energy of 38 mN m^−1^. We demonstrated the fabrication of exceptionally high-performance, hysteresis-free n-type solution-processed oxide transistors comprising an In_2_O_3_/ZnO double layer as an active channel with an electron mobility of over 50 cm^2^ V^−1^ s^−1^, on/off ratio of ~10^7^, subthreshold swing of 108 mV dec^−1^, and high bias-stress stability. From temperature-dependent *I–V* analyses combined with charge transport mechanism analyses, we demonstrated that the proposed hybrid dielectric layer provides percolation-limited charge transport for the In_2_O_3_/ZnO double layer under field-effect conditions.

## Abstract (web summary)

Low cost and low power consumption of transistors are needed for the development of internet of things. Here Chung et al. develop a high performance n-type oxide thin film transistor by introducing a ligand for crosslinking nanoparticles and polymers, obtaining a near-ideal hybrid dielectric layer.

## Introduction

Owing to the global development in IoT technology, interest in metal-oxide semiconductor-based circuits with low standby power consumption, particularly in thin-film transistor (TFT) materials capable of low-cost solution processing, has been rapidly increasing^1–3^. Among several solution-processable semiconductors, metal oxides are regarded as the most successful material platforms for TFT mainly because of their high charge carrier mobility and operational stability^4–6^. Recently, Yang et al. reported a high-performance solution-processed oxide TFT with high electron mobility under UV irradiation^7^. Using UV/ozone treatment prior to thermal annealing, Shan et al. demonstrated a low subthreshold swing with low operating voltage facilitating a dense and smooth surface^8^. In addition, Anthopoulos et al. reported a high-performance solution-processed oxide TFT with excellent operational stability by passivating the electron traps in the channel via the insertion of an ozone-treated polymer interlayer^9^.

Both the oxide semiconductor layer and gate dielectric layer significantly influence the oxide TFT performance. Notably, most of the high electron mobility (>30 cm^2^ V^−1^ s^−1^) oxide TFTs were based on high-*k* inorganic gate dielectrics. Compared with conventional SiO_2_ (*k* = 4), high-*k* oxides such as HfO_2_, ZrO_2_, Ta_2_O_5_, and Al_2_O_3_ are ideal candidates for realizing a high-capacitance dielectric layer capable of low-voltage driving and high density of charge accumulation^10–14^. However, these high-*k* inorganic dielectric layers are hindered by (1) expensive deposition equipment and high process temperature for vacuum deposition and (2) imperfect inorganic purity and porous morphology for solution deposition^15^. For oxide TFTs with solution-deposited high-*k* dielectric layers, high electron mobility, high on/off ratio, and low subthreshold swing have rarely been simultaneously achieved. Therefore, considering the successful commercialization of all-solution-processed oxide TFTs, the development of a solution-processed high-*k* dielectric layer with high dielectric strength is urgently required. A possible strategy to strengthen the dielectric properties of solution-processed high-*k* oxides is to realize an organic–inorganic hybrid dielectric layer, which typically comprises nanocomposites of polymer and high-*k* oxide nanoparticles (NPs) combining high permittivity of the inorganic NPs and high breakdown strength, mechanical flexibility, and easy processability of the polymer dielectrics^16^. However, existing hybrid dielectric layers have not shown this synergistic effect and yielded only marginal performances, particularly for oxide transistors.

The aforementioned synergetic effects of ideal organic–inorganic hybrid dielectric layers can be achieved only when the complementary organic and inorganic constituents are well mixed, and the resulting thin film is defect-free. In reality, owing to the different surface energies of organic and inorganic inclusions, the thin-film morphology of hybrid dielectrics often has the limitations of air voids and low thin-film density, thereby resulting in a high leakage current and consequently a low on/off ratio of the resulting TFT^17^. Furthermore, owing to the thermodynamic instability of the mixed binary phase, the hybrid dielectric layer can lack long-term operational stability. In fact, the oxide TFTs with hybrid dielectric layers reported thus far only rendered either (1) overall low performance or (2) high electron mobility, but with low on/off ratios or high subthreshold swing. These results indicate that it is challenging to simultaneously realize a high-*k* and high dielectric strength from a hybrid dielectric layer.

**Fig. 1:**
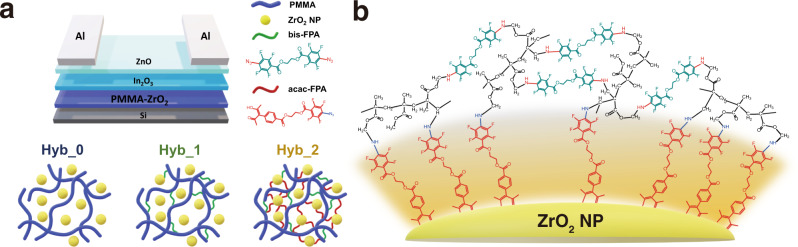
Schematic diagram of oxide TFT with hybrid dielectric using a crosslinking ligand. **a** Structure of In_2_O_3_/ZnO heterojunction TFT with PMMA-ZrO_2_ as a gate dielectric (top) and molecular structure of Hyb_0, Hyb_1, and Hyb_2 (bottom). **b** Proposed structure of ZrO_2_ NPs with acac-FPA as a chelating ligand and binding with polymer via azide chemistry whereas bis-FPA effectively crosslink polymeric backbone.

In this study, we attempted to achieve an organic–inorganic hybrid dielectric layer with a covalently networked morphology between organic and inorganic inclusions for high-performance solution-processed oxide transistors. As an organic inclusion, poly(methyl methacrylate) (PMMA) which exhibits high dielectric strength, thermal and chemical stability, facile thin film fabrication process, and feasibility of singlet nitrene-mediated cross-linking reaction was used^4^. As an inorganic inclusion, ZrO_2_ which simultaneously satisfies key requirements for high-*k* materials such as high dielectric constant, wide energy bandgap, high thermal stability, and high breakdown field was used^18,19^. Therefore, we propose a method for chemically crosslinking zirconia NPs and PMMA with functionalized azide ligands. Compared with previous crosslinking methods for hybrid dielectrics with existing coupling agents, azide chemistry has a significantly higher crosslinking efficiency with a minimized amount of crosslinker, which results in a higher dielectric constant^20^. However, simply mixing azide molecules with ZrO_2_ NPs and PMMA cannot guarantee crosslinking between inorganic and organic phases; nitrene generated from azide can react with alkyl CH or *π*-conjugated aromatic groups; therefore, crosslinking is limited only between PMMAs and not between the ZrO_2_ NPs and PMMA. Therefore, we synthesized azide-functionalized acetylacetonate, functioning as a (1) ligand of ZrO_2_ NP in post sol–gel synthesis and (2) crosslinking agent between ZrO_2_ NP and PMMA (Fig. 1). The processing conditions for the proposed hybrid dielectric layer were optimized, which demonstrated defect-free, near-ideal dielectric performance with a dielectric strength of over 4.0 MV cm^−1^, the surface energy of ~38 mN m^−1^, and dielectric constant of ~14. The synergetic contributions of the hybrid dielectric layer enabled the demonstration of exceptionally high-performance n-type oxide transistors consisting of an In_2_O_3_/ZnO double layer as an active channel, whose charge transport mechanism was found percolation-limited.

## Results

### Synthesis and characterization of acac-FPA and bis-FPA

Several chelating ligands, such as alcohols, diols, carboxylic acids, silanes, boric acids, and phosphonates, are used to attach coordination complexes (dyes) to metal-oxide surfaces, for photocatalytic research^21^. Among these, carboxylic acids are commonly used because of their strong covalent bonding with the metal-oxide surface. Batista et al. reported acetylacetonate (acac), which is a bidentate ligand, as a chelating agent for the robust functionalization of Mn(II)–terpyridine complexes and TiO_2_ NPs^22^. Acac is known for its stability under humid and oxidative conditions which is superior to those of conventional carboxylate groups. Several types of chelating ligands have been reported to form coordination complexes with zirconium ions, with acac forming a more hydrolytically stable complex. Although several reports have demonstrated the successful functionalization of ZrO_2_ NPs surfaces with acac moieties^23–25^, the functionalization of these acac scaffolds with crosslinking agents has not been attempted yet.

**Fig. 2:**
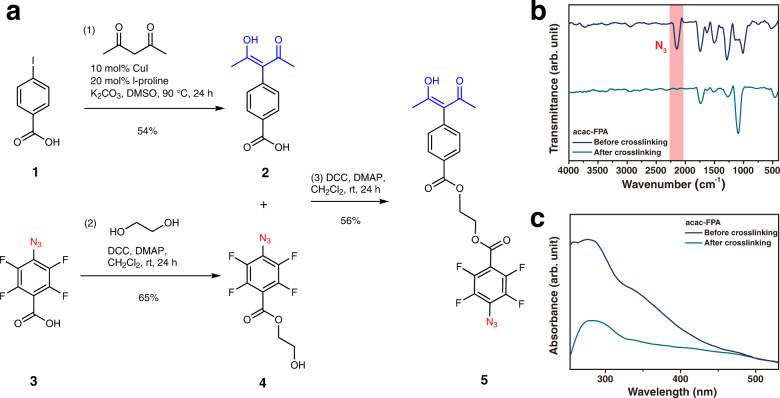
Synthetic scheme and characterization of acac-FPA as a chelating ligand. **a** Synthetic procedure for acac-FPA. **b** FT-IR spectra of acac-FPA films before and after being cross-linked. The red shade represents the presence of azide groups via the asymmetric stretching band *ν*_as_ (N_3_). **c** UV–visible absorption spectra of acac-FPA films before and after being cross-linked.

In this study, we developed an acac scaffold, 2-((4-(1-acetyl-2-hydroxy-1-propen-1-yl)benzoyl)oxy)ethyl 4-azido-2,3,5,6-tetrafluorobenzoate (acac-FPA), in which the 2,4-pentadionate linker was substituted with an azide at the central bond, as shown in Fig. 2a, which can successfully bind to the ZrO_2_ NP surface as well as crosslink with the polymer. For more control over the hydrolysis and condensation reactions of inorganic oxide sols, the acac ligand was selected, because a weak binding ligand leads to unstable and larger particle sizes in the sols than in those with strong binding ligands.

For crosslinking, aryl azide was selected because it generates reactive nitrenes upon UV light irradiation, which can initiate insertion reactions with C–H or N–H bonds, addition reactions with double bonds of neighboring molecules, or subsequent ring expansion, predominantly in the presence of primary amines to form stable covalently linked moieties^26^. Recently, the fluorophenyl azide (FPA) moiety has garnered significant attention owing to its efficient generation of singlet nitrenes that can be inserted into unactivated C–H bonds^27–31^. Non-fluorinated phenyl azides and alkyl azides provide lower photocrosslinking efficiencies owing to the short lifetime of singlet nitrenes^26^. Therefore, in this study, the FPA moiety was incorporated with an acac moiety to efficiently crosslink the polymer with metal-oxide NPs.

The chemical structure of acac-FPA, consisting of FPA and an acac moiety acting as a crosslinker and chelating ligand, respectively, is depicted in Fig. 2a. For reference, ethane-1,2-diyl bis(4-azido-2,3,5,6-tetrafluorobenzoate) (bis-FPA), which has two FPA groups and can serve as only a photocrosslinker, was synthesized^32^. Both of these molecules were chosen for the following reasons: (1) structural analogs owing to the presence of a similar azide moiety, (2) facile synthesis owing to the available starting materials, and (3) ease of characterization, arising from distinct IR absorption peaks of azide. Acac-FPA was prepared in favorable yields using acetylacetone. FPA-acac was prepared via the Hurtley reaction between 4-iodobenzoic acid and acetylacetone, followed by dicyclohexyl carbodiimide (DCC) coupling between 3-(4-benzoic acid)pentane-2,4-dione and 2-hydroxyethyl 4-azido-2,3,5,6-tetrafluorobenzoate, as shown in Fig. 2a. Bis-FPA was prepared via DCC coupling between ethylene glycol and 4-azido-2,3,5,6-tetrafluorobenzoic acid^32,33^. Bis-FPA and acac-FPA are soluble in methanol, ethanol, 2-methoxyethanol, and aprotic solvents, including acetonitrile, acetone, dichloromethane, chloroform, dimethylformamide (DMF), and DMSO; hence, such photocrosslinkers can be blended with various types of polymers in appropriate solvents. Moreover, because of the favorable shelf life of these crosslinkers, they can be stored under dark and vacuum conditions for over a year.

The Fourier transform infrared spectroscopy (FT-IR) spectra of bis-FPA and acac-FPA confirmed the presence of azide groups via the asymmetric stretching band *ν*_as_ (N_3_) in the 2000–2200 region as shown in Supplementary Fig. 1 and Fig. 2b, respectively^26^. The UV–vis absorption spectra of bis-FPA and acac-FPA show characteristic peaks of the FPA moiety in the range of 250–320 nm which subsequently reduce in intensity after UV light irradiation, as shown in Supplementary Fig. 2 and Fig. 2c, respectively.

The PMMA–ZrO_2_ hybrid was prepared by mixing PMMA, Zr(acac)_4_, and an appropriate amount of crosslinker bis-FPA and acac-FPA in a DMF solvent. Notably, when Zr(acac)_4_ is used for the synthesis of ZrO_2_ NPs, some chelating ligands remain on the surface, which can undergo ligand exchange with various types of ligands^34,35^; thus, this strategy of ligand exchange was adopted so that an adequate amount of acac-FPA could bind to the ZrO_2_ NP surface. To investigate the effects of crosslinking and chelating ligands on the PMMA–ZrO_2_ hybrid, three different hybrid films were prepared: PMMA/ZrO_2_, PMMA/ZrO_2_/bis-FPA with 5 wt% bis-FPA, and PMMA/ZrO_2_/bis-FPA/acac-FPA with 5 wt% bis-FPA and acac-FPA, named Hyb_0, Hyb_1, and Hyb_2, respectively. Note that PMMA/ZrO_2_/bis-FPA with 10 wt% of bis-FPA gave very similar characteristics compared to Hyb_1 with 5 wt% of bis-FPA, in terms of dielectric strength as shown in Supplementary Fig. 3, and here we compared only Hyb_1 with other hybrid dielectrics. The asymmetric stretching at 2093 and 2094 in the FT-IR spectra of the Hyb_1 and Hyb_2 hybrids, respectively, indicates the presence of azide, which diminishes after UV light irradiation, as shown in Supplementary Fig. 4, indicating successful crosslinking within the thin film. Note that the IR stretching of the C=O bond at 1733 cm^−1^ present in Hyb_1 and Hyb_2 is likely because of the presence of C=O in either bis-FPA, acac-FPA, or PMMA. Thus, there was no difference in the C=O stretching of Hyb_1 and Hyb_2 as the weight ratio of bis-FPA and acac-FPA is significantly low (~5 wt%) with respect to the PMMA/ZrO_2_ hybrid.

### Characterization of hybrid dielectrics

**Fig. 3:**
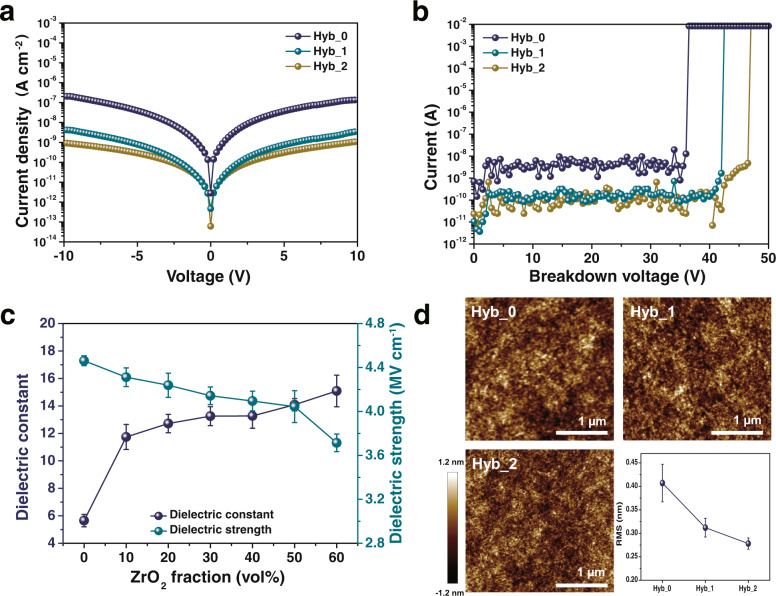
Characterizations of hybrid dielectric layers. **a** Gate leakage current density versus applied voltage. **b** Breakdown voltage. **c** Dielectric constant and dielectric strength as a function of ZrO_2_ loading ratios. Error bars represent the standard deviation from 10 devices. **d** AFM topography images and rms roughness values: 0.407, 0.312, and 0.278 nm for Hyb_0, Hyb_1, and Hyb_2, respectively. Error bars represent the standard deviation from 10 devices.

To optimize the mixing ratios of PMMA and sol–gel-synthesized ZrO_2_ NP with acac-FPA and bis-FPA, capacitance measurements were conducted with the metal–insulator–metal (MIM) structure by varying the mixing ratios. MIM capacitors were fabricated on bare Si substrates by spin-coating the hybrid solution followed by photocrosslinking. The *J–V* characteristics of the MIM capacitors are plotted as a function of the voltage (Fig. 3a). Hyb_0 and Hyb_1 exhibited leakage current values of 1.37 × 10^−7^ and 6.54 × 10^−9^ A cm^−2^ at 10 V, respectively. In contrast, Hyb_2 showed a lower leakage current value of 9.45 × 10^−10^ A cm^−2^ at 10 V. Hyb_0 and Hyb_1 exhibited breakdown voltages of 36.0 and 42.0 V, respectively. In contrast, Hyb_2 exhibited a higher breakdown voltage of 46.5 V (Fig. 3b). The areal capacitance values measured at 1 kHz and the breakdown voltage were recorded, which evidently increase and decrease, respectively, with increasing ZrO_2_ NP loading ratios as shown in Supplementary Fig. 5. In addition, SEM cross-section image and the changes of dielectric properties as a function of the thickness are shown in Supplementary Figs. 6 and 7, respectively. The thickness of the hybrid dielectric layer is 115 nm, which was chosen considering the optimal capacitance and dielectric strength.

Then, the relative permittivity or dielectric constant (*k*) was calculated using the following equation^36^:

1 $$C={\varepsilon }_{0}{\varepsilon }_{{{{{{\rm{r}}}}}}}\frac{A}{d},$$

where *C* is the capacitance of the dielectric, *ε*_0_ is the permittivity of a vacuum, *ε*_r_ is the relative permittivity of the dielectric, *A* is the area, and *d* is the thickness of the dielectric layer. The PMMA and ZrO_2_ precursor solutions were blended at different volumetric ratios (PMMA:ZrO_2_, vol%) of 100:0 (Zr-0), 90:10 (Zr-10), 80:20 (Zr-20), 70:30 (Zr-30), 60:40 (Zr-40), 50:50 (Zr-50), and 40:60 (Zr-60). The obtained dielectric constants (*k*) for the Zr-0, Zr-10, Zr-20, Zr-30, Zr-40, Zr-50, and Zr-60 dielectric films were 5.65, 11.74, 12.72, 13.26, 13.27, 14.09, and 15.09, respectively, reflecting the loading effect of ZrO_2_ NPs into the PMMA matrix (Fig. 3c). In contrast, the dielectric strengths for the Zr-0, Zr-10, Zr-20, Zr-30, Zr-40, Zr-50, and Zr-60 dielectric films were 4.46, 4.31, 4.23, 4.14, 4.09, 4.04, and 3.71 MV cm^−1^, respectively, which was attributable to the presence of pinholes and the rough morphology in the cases of high ZrO_2_ NPs loading ratios. (Fig. 3c) Here, the dielectric strength is defined as the maximum electric field that the dielectric can endure, as shown in Fig. 3b. With respect to dielectric breakdown, free electrons in the conduction band of dielectrics with large fields can collide with the host atoms in the solid and be accelerated to energies sufficiently high to ionize. If voids are present in the dielectric, the electric field applied to the void increases with the ratio of *ε*_d_/*ε*_v_ where *ε*_v_ and *ε*_d_ are the dielectric constants of air voids and dielectric media, respectively, making them vulnerable to impact ionization^36^. Therefore, decreasing the dielectric strength with increasing Zr loading ratio indicates an increase in the number of air voids created by inorganic inclusions within the polymer matrix. When the volume ratio of PMMA and ZrO_2_ NPs was 50:50, both the dielectric constant and dielectric strength were reasonably high, and this was considered an optimized condition.

The effective dielectric constants of various types of dielectrics with dispersed pores can be predicted using the Maxwell–Garnett equation^36^:

2 $$\frac{{\varepsilon }_{{{{{{\rm{reff}}}}}}}-{\varepsilon }_{{{{{{\rm{r}}}}}}2}}{{\varepsilon }_{{{{{{\rm{reff}}}}}}}+2{\varepsilon }_{{{{{{\rm{r}}}}}}2}}={v}_{1}\frac{{\varepsilon }_{{{{{{\rm{r}}}}}}1}-{\varepsilon }_{{{{{{\rm{r}}}}}}2}}{{\varepsilon }_{{{{{{\rm{r}}}}}}1}+2{\varepsilon }_{{{{{{\rm{r}}}}}}2}},$$

where *ε*_reff_ is the effective dielectric constant, *ε*_r1_ and *ε*_r2_ are the dielectric constants, and *v*_1_ and *v*_2_ are the volume fractions of mixed phases I and II, respectively. In the optimized condition, dielectric constants of pristine PMMA and ZrO_2_ NPs dielectric layer were measured as 5.65 and 24.44, respectively. The effective dielectric constants of PMMA and ZrO_2_ NPs were calculated as 4.53 and 22.14, respectively, using the effective dielectric constant equation^37^:

3 $${\varepsilon }_{{{{{{\rm{reff}}}}}}}=\frac{{\varepsilon }_{{{{{{\rm{r}}}}}}}+1}{2}+\frac{{\varepsilon }_{{{{{{\rm{r}}}}}}}-1}{2}\frac{1}{\sqrt{1+12\frac{h}{W}}},$$

where *h* is the thickness of the dielectric layer and *W* is the width of the patch. Therefore, the porosities of the PMMA and ZrO_2_ NPs can be calculated from Eq. (2) as 18.7% and 6.9%, respectively. In addition, the porosities of the Hyb_0, Hyb_1, and Hyb_2 dielectric layers can be obtained using Lichtenecker’s equation^36,38^:

4 $${{{{{\rm{ln}}}}}}{\varepsilon }_{{{{{{\rm{reff}}}}}}}={v}_{1}{{{{{\rm{ln}}}}}}{\varepsilon }_{{{{{{\rm{r}}}}}}1}+{v}_{2}{{{{{\rm{ln}}}}}}{\varepsilon }_{{{{{{\rm{r}}}}}}2}+{v}_{3}{{{{{\rm{ln}}}}}}{\varepsilon }_{{{{{{\rm{r}}}}}}3},$$

where *ε*_reff_ is the effective dielectric constant of the hybrid dielectric layer; *ε*_r1_, *ε*_r2_, and *ε*_r3_ are the dielectric constants; and *v*_1_, *v*_2_, and *v*_3_ are the volume fractions of PMMA, ZrO_2_ NPs, and air pores, respectively. Here volume fractions were calculated using the weight and density of each constituent. The porosities of the Hyb_0, Hyb_1, and Hyb_2 dielectric layers are 4.4%, 2.0%, and 1.4%, respectively. In addition, the surface morphology and roughness of the PMMA–ZrO_2_ dielectric films were analyzed using atomic force microscopy (AFM) (Fig. 3d) at a scan area of 1 μm × 1 μm with different crosslinking agents. The measured root-mean-square (rms) values of Hyb_0, Hyb_1, and Hyb_2 dielectric layers of 0.407, 0.312, and 0.278 nm, respectively, indicating that using acac-FPA as both the chelating ligand and crosslinking agent was an effective approach for dense and void-free hybrid dielectric layers.

In addition, to investigate the effect of different crosslinkers on the surface free energy of the PMMA–ZrO_2_ hybrid dielectric layer, the contact angle was measured (Supplementary Fig. 8) and is summarized in Supplementary Table 1. Hyb_2 has the lowest surface energy of 38.39 mN m^−1^, which is lower than 47.51, 58.77, and 39.55 mN m^−1^ of crosslinked-PMMA (PMMA blended with 5 wt% of bis-FPA), Hyb_0, and Hyb_1, respectively, indicating that acac-FPA crosslinks better with ZrO_2_ NPs to reduce porosity and facilitate a hydrophobic surface.

The distribution and particle shape of ZrO_2_ in Hyb_2 were analyzed using scanning electron microscope-energy dispersive X-ray spectrometer (SEM-EDS) and transmission electron microscope (TEM). As shown in SEM-EDS analysis, the Hyb_2 renders a defect-free surface and even distribution of PMMA and ZrO_2_ throughout the thin film (Supplementary Fig. 9). In addition, TEM analysis shows that ZrO_2_ clusters are well distributed within the matrix PMMA over the entire dielectric surface with cluster size <100 nm (Supplementary Fig. 10).

To confirm the presence of ZrO_2_ in Hyb_2, the chemical compositions of Hyb_2 were investigated by X-ray photoelectron spectroscopy (XPS) measurement (Supplementary Fig. 11). The survey scan shows photoelectron peaks at binding energies of 532.1, 284.4, and 183.6 eV, which were associated with the O 1*s*, C 1*s*, and Zr 3*d*, respectively (Supplementary Fig. 11a). The deconvoluted Zr 3*d* peaks with spin–orbit doublets of 183.6 and 186.2 eV separated by 2.6 eV indicate the formation of ZrO_2_ (Supplementary Fig. 11c)^39^. Especially, the peaks at 531.4 and 533.2 eV were attributed to M–O–M and M–OH species, respectively, which means that ZrO_2_ with Zr–O–Zr bond exists in the Hyb_2 (Supplementary Fig. 11d)^40^.

Collectively, SEM, TEM, and XPS analyses suggest that Hyb_2 is a near-ideal organic–inorganic hybrid film consisting of small-sized clusters of ZrO_2_ with even distribution, resulting in excellent dielectric strength and high dielectric constant.

### *I*–*V* characteristics of heterojunction TFT

**Fig. 4:**
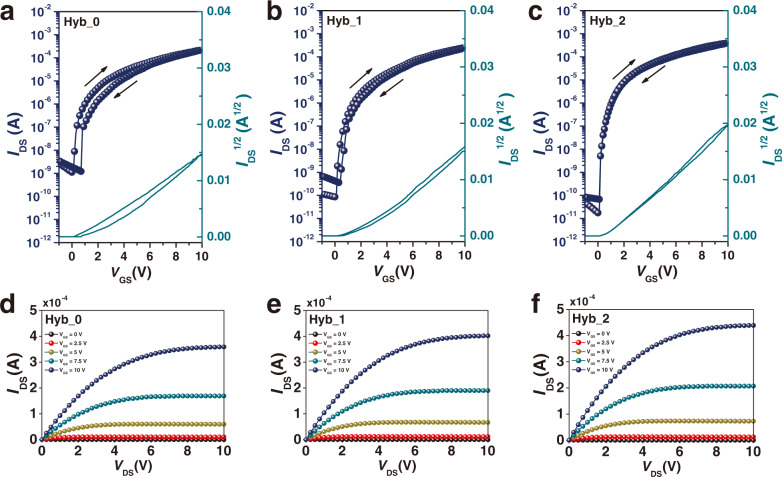
Current–voltage characteristics of In_2_O_3_/ZnO heterojunction TFTs. Representative transfer characteristics of the TFTs with **a** Hyb_0, **b** Hyb_1, and **c** Hyb_2 as gate dielectrics, measured at *V*_D_ = 10 V. Output characteristics of the TFTs with **d** Hyb_0, **e** Hyb_1, and **f** Hyb_2 as gate dielectrics. The gate voltage was increasingly varied between 0 and 10 V in steps of 2.5 V.

The performance of the synthesized dielectric film as a gate dielectric was evaluated by fabricating In_2_O_3_/ZnO heterojunction TFTs. One of the key mechanisms for increasing the charge mobility of oxide TFT is the effective formation of charge accumulation. In particular, in the heterojunction between an oxide upper layer with a low work function (e.g., ZnO) and an oxide lower layer with a high work function (e.g., In_2_O_3_), electron diffusion causes electrons to accumulate along the boundary between the two regions. Therefore, In_2_O_3_/ZnO heterojunction TFTs use the principle that the electron accumulation at these interfaces leads to high currents. The representative transfer characteristics with Hyb_0, Hyb_1, and Hyb_2 are shown in Fig. 4a–c, respectively, exhibiting electron mobilities of 30.93, 42.32, and 51.19 cm^2^ V^−1^ s^−1^, on/off ratios of ~10^5^, ~3 × 10^6^, and ~10^7^, threshold voltages of 0.75, 0.59, and 0.46 V, subthreshold swings of 296, 156, and 108 mV dec^−1^, and trap concentration per unit energy of 2.66 × 10^12^, 1.08 × 10^12^, and 5.42 × 10^11^ eV^−1^ cm^−2^, respectively, implying that the strategy of Hyb_2 is effective for suppressing trap states. (Table 1) This demonstrates a significantly superior performance to that of the previously reported TFT with a high-*k* oxide and hybrid dielectric. (Supplementary Table 2)^41^ In Fig. 4d–f, *I*_DS_ linearly increases with increasing *V*_DS_ in the initial linear regime, and *I*_DS_ is saturated at the high *V*_DS_ regimes for overall *V*_GS_, which means a pinch-off of the channel near the drain end at high *V*_DS_. Note that such a pinch-off can be observed only when the dielectric layer can induce even charge accumulation at the channel region at a given gate dielectric^42,43^.

**Table 1:** Electrical characteristics of In_2_O_3_/ZnO heterojunction TFTs with Hyb_0, Hyb_1, and Hyb_2 as gate dielectrics

Dielectric layer	Active layer	Mobility (cm^2^ V^−1^ s^−1^)	*I*_on_/*I*_off_	*V*_TH_ (V)	SS (mV dec^−1^)	*D*_tr_ (eV^−1^ cm^−2^)
Hyb_0	In_2_O_3_/ZnO	30.93 ± 7.29	~10^5^	0.75	296	2.66 × 10^12^
Hyb_1	In_2_O_3_/ZnO	42.32 ± 4.24	~3 × 10^6^	0.59	156	1.08 × 10^12^
Hyb_2	In_2_O_3_/ZnO	51.19 ± 3.13	~10^7^	0.46	108	5.42 × 10^11^

In addition, In_2_O_3_/ZnO TFTs with Hyb_2 fabricated under optimized conditions showed high device reproducibility, with an average electron mobility of ~51 cm^2^ V^−1^ s^−1^ calculated from 50 independently fabricated devices (Supplementary Fig. 12).

### Bias-stress stability of heterojunction TFT

**Fig. 5:**
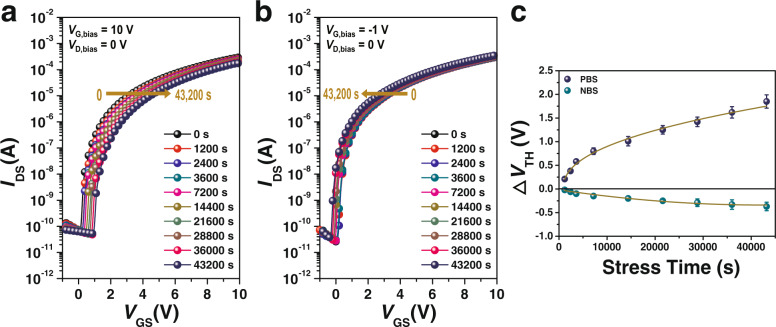
Operational bias-stress stability of In_2_O_3_/ZnO heterojunction TFTs with Hyb_2 as a gate dielectric. **a** Transfer characteristics under PBS (*V*_G,bias_ = 10 V, *V*_D,bias_ = 0 V). **b** Transfer characteristics under NBS (*V*_G,bias_ = −1 V, *V*_D,bias_ = 0 V). The bias-stress time varied between 0 and 43,200 s. **c** Time dependence of △*V*_TH_ as a function of stress time under PBS and NBS conditions. Error bars represent the standard deviation from 10 devices.

Provided the high electron mobility, In_2_O_3_/ZnO heterojunction TFTs with Hyb_2 are expected to exhibit favorable bias-stress stability owing to their reduced trap concentration. We subjected the TFT to continuous positive and negative bias-stress of *V*_G,bias_ = 10 and −1 V, respectively, for 12 h in a dark environment, and the results of the bias-stress stability for the In_2_O_3_/ZnO heterojunction TFTs with Hyb_2 are summarized in Fig. 5. Under the positive bias-stress (PBS), the bias-stress stability-induced shift in *V*_TH_ (△*V*_TH_) was relatively low (~1.8 V) despite the long stress time (Fig. 5a). The PBS stability of TFT is generally considered to be owing to electron trapping at the interface between the channel layer and the dielectric layer or bulk dielectric layer, yielding a positive △*V*_TH_^44^. We quantitatively analyzed the bias-stress stability results by fitting the time (*t*) dependence of *V*_TH_ to a stretched exponential equation:

5 $$\left|\bigtriangleup {V}_{{{{{{\rm{TH}}}}}}}\right |={V}_{0}\left[1-{{\exp }}\left\{-{\left(\frac{t}{\tau }\right)}^{{{{{{\rm{\beta }}}}}}}\right\}\right],$$

where *V*_0_ is the saturation value of $\left|\bigtriangleup {V}_{{{{{{\rm{TH}}}}}}}\right|$, $\tau={\tau }_{0}{{\exp }}(\frac{{E}_{{{{{{\rm{\tau }}}}}}}}{kT})$ is the characteristic time of carrier trapping given the thermal activation energy as ${E}_{{{{{{\rm{a}}}}}}}={E}_{{{{{{\rm{\tau }}}}}}}\beta$, and *β* is the stretched exponential parameter with a numerical value in the range 0 < *β* ≤ 1^9^ (Fig. 5b). A stretching parameter close to 1 indicates a narrow distribution of time constants, whereas a stretching parameter close to 0 indicates that the time taken for $\left|\bigtriangleup {V}_{{{{{{\rm{TH}}}}}}}\right|$ to reach saturation is significantly long. The obtained fitting parameters are *τ* = 4.06 × 10^5^ s and *β* = 0.64. The low $\left|\bigtriangleup {V}_{{{{{{\rm{TH}}}}}}}\right|$ and *τ* values combined with high *β* values indicate that In_2_O_3_/ZnO heterojunction TFTs with Hyb_2 exhibit a shallower and narrower trap distribution, as well as a lower trap state concentration, which is ascribable to the excellent dielectric properties of Hyb_2 and the efficient electron accumulation property of the In_2_O_3_/ZnO heterojunction. Considering that most of the previously reported studies on the bias-stress stability of oxide TFTs with high-*k* dielectrics have monitored stress times up to 3600 s, we also attempted to extract fitting parameters under identical conditions, and the results were *τ* = 6.07 × 10^4^ s and *β* = 0.94. Therefore, we argue that the proposed hybrid dielectric strategy enables superior bias-stress stability compared with other high-*k* oxide dielectrics. In the case of negative bias-stress (NBS), △*V*_TH_ is known to be related to trapped holes and/or the accumulation of positive charge along the increase of the stress time, resulting in negative value^45,46^ (Fig. 5b, c). In the case of Hyb_2, −0.48 V of △*V*_TH_ was measured after the 43,200 s NBS test, which is comparable to that of TFT with SiO_2_ dielectric (Supplementary Table 3). Under both PBS and NBS conditions, a low trap concentration of 5–6 × 10^11^ eV^−1^ cm^−2^ was well-maintained in the case of Hyb_2, indicating the strategy of using acac-FPA is universally efficient for suppressing trap states (Supplementary Table 4).

### Temperature dependence of heterojunction TFT

**Fig. 6:**
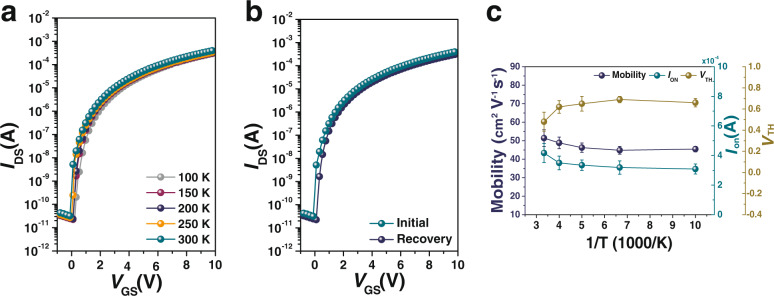
Temperature-dependent characteristics of In_2_O_3_/ZnO heterojunction TFTs with Hyb_2 as a gate dielectric. Temperature-dependent transfer curve **a** for the measurement temperature increased from 100 to 300 K in steps of 50 K and **b** for comparison of transfer curves at room temperature before and after the temperature-dependent measurement. **c** Arrhenius plots of the electron mobility, on current, and threshold voltage. Error bars represent the standard deviation from 10 devices.

To elucidate the physical reason for the success of the In_2_O_3_/ZnO heterojunction TFT with a Hyb_2 dielectric layer, temperature-dependent transfer characteristics were measured, as summarized in Fig. 6a, from 100 to 300 K in steps of 50 K. With increasing temperature, the transfer curve shifted slightly in the negative gate voltage direction, and the on and off currents increased. The transfer characteristics of the In_2_O_3_/ZnO TFT were measured at room temperature before and after temperature-dependent measurements, as shown in Fig. 6b. The transport properties at room temperature were mostly reproduced after the temperature-dependent measurement, although it is not ideal, indicating that the effect of oxygen vacancies due to thermal excitation was low, and a few defects between the dielectric layer and the channel layer were present^47^. To further understand the carrier transport mechanism of the In_2_O_3_/ZnO TFT, Arrhenius plots of electron mobility, on current, and *V*_TH_ are shown in Fig. 6c. The In_2_O_3_/ZnO TFT exhibited temperature-activated behavior. The electron mobility remains constant (46–48 cm^2^ V^−1^ s^−1^) within the low-temperature region of 100 K ≤ *T* ≤ 200 K, and slightly increases within the high-temperature region of 200 K ≤ *T* ≤ 300 K with a maximum of ~51 cm^2^ V^−1^ s^−1^ at room temperature. In the Arrhenius model, the drain current can be expressed using the following equation^36^:

6 $$\mu={\mu }_{0}\cdot {{\exp }}\left(-\frac{{E}_{{{{{{\rm{a}}}}}}}}{{k}_{{{{{{\rm{B}}}}}}}T}\right),$$

where $\mu$_0_ is the initial mobility, *E*_a_ is the activation energy, and *k*_B_ is Boltzmann’s constant. *E*_a_ is the energy difference between the minimum value of the conduction band, *E*_C_, and the Fermi level, *E*_F_, which is the energy required to emit an electron from a localized state. The activation energy, *E*_a_, determined from the 200 K ≤ *T* ≤ 300 K region was ~5.4 meV, as shown in Supplementary Fig. 13, indicating a practically negligible trap state density compared with other oxides TFTs or near-ideal percolation-limited charge transport^48^.

### Carrier transport mechanism

To more quantitatively confirm the carrier transport mechanism, the relative dominance of trap-limited and percolation-limited conductions was investigated in In_2_O_3_/ZnO heterojunction TFTs with Hyb_2. When the Fermi energy level (*E*_F_) is in a significantly lower energy state than the conduction band edge (*E*_m_) at the limit of low gate voltage, the carrier transport is controlled by trap-limited conduction. In contrast, when *E*_F_ lies on *E*_m_ at a high gate voltage, the tail states are filled, and thus, carrier transport is controlled by percolation. Therefore, *n*_free_ and *n*_trap_ were calculated using Fermi–Dirac statistics as a function of the gate voltage to quantitatively estimate the carrier transport mechanism. The ratio of the free carrier density of the interface at the driving voltage can be estimated using the following equations^49^:

7 $${n}_{{{{{{\rm{free}}}}}}}\left({E}_{{{{{{\rm{F}}}}}}}\right)=\left[\frac{{\left(2{m}_{{n}}^{*}\right)}^{\frac{3}{2}}}{2{\pi }^{2}{\hbar }^{3}}\right]{\int }_{{E}_{{{{{{\rm{m}}}}}}}}^{\infty }\sqrt{E-{E}_{{{{{{\rm{m}}}}}}}}\,f\left(E\right){{{{{{\rm{d}}}}}}E},$$

8 $${n}_{{{{{{\rm{trap}}}}}}}\left({E}_{{{{{{\rm{F}}}}}}}\right)={\int }_{{E}_{{{{{{\rm{F}}}}}}0}}^{{E}_{{{{{{\rm{m}}}}}}}}{N}_{{{{{{\rm{t}}}}}}}(E)\,f\left(E\right){{{{{{\rm{d}}}}}}E},$$

where ${m}_{{{{{\rm{n}}}}}}\!\!*$ is the effective mass of the electron, $\hbar$ is the modified Planck’s constant; ${N}_{{{{{{\rm{t}}}}}}}\left(E\right)={N}_{{{{{{\rm{tc}}}}}}}{{\exp }}((E-{E}_{{{{{{\rm{m}}}}}}})/k{T}_{{{{{{\rm{t}}}}}}})$ and $f\left(E\right)={(1+{{\exp }}\left\{(E-{E}_{{{{{{\rm{F}}}}}}})/{kT}\right\})}^{-1}$. The gate voltage dependence of mobility can be obtained using the power law equation:

9 $${\mu }_{{{{{{\rm{FE}}}}}}}={K\left({V}_{{{{{{\rm{G}}}}}}}-{V}_{{{{{{\rm{T}}}}}},{{{{{\rm{P}}}}}}}\right)}^{{{{{{\rm{\gamma }}}}}}},$$

where prefactor *K* and exponent *γ* is related to the properties of the transport mechanism. In the range of low gate voltage, *γ* and *kT*_t_ of 0.74 and 0.035, respectively, can be obtained using Supplementary Fig. 14a, which shows the drain current and electron mobility as a function of gate voltage. Detailed calculation methods have been reported elsewhere^49,50^. Therefore, *n*_free_/(*n*_free_ + *n*_trap_) at the driving voltage was 0.96 (Supplementary Fig. 14b), indicating that the carrier transport mechanism is mainly dominated by percolation, which agrees well with the temperature-dependent measurements. The same analyses were conducted on In_2_O_3_/ZnO TFTs with a SiO_2_ dielectric layer, and *n*_free_/(*n*_free_ + *n*_trap_) is observed to be 0.74, as shown in Supplementary Fig. 15. Therefore, we propose that the combination of the Hyb_2 dielectric layer with the In_2_O_3_/ZnO heterojunction strategy enables a more ideal charge transport of oxide TFTs.

### Superiority of hybrid dielectric with acac-FPA

The electron conduction in the amorphous oxide field-effect transistor is controlled by percolation and trap-limited conduction. Typically, deriving percolation-limited conduction is crucial for realizing high electron mobility. In particular, for low-temperature solution-processed oxides, few reports demonstrate a percolation-dominated conduction process. Anthopoulos et al. reported a high electron mobility of 45 cm^2^ V^−1^ s^−1^ for an In_2_O_3_/ZnO heterojunction TFT with SiO_2_ as the gate dielectric layer^48^. In this study, an almost temperature independence of the linear mobility was observed, indicative of trap-free band-like electron transport or percolation-limited conduction. Although a few more studies on electron mobility over 50 cm^2^ V^−1^ s^−1^ from low-temperature solution processes were reported, it is noteworthy that all these reports were in conjunction with conventional SiO_2_ or high-*k* inorganic dielectric layers. These oxide TFTs using high-*k* dielectrics exhibited high electron mobility, but this is usually accompanied by a low on/off ratio and high subthreshold swing owing to the high leakage current. Realizing genuine high-performance or percolation-limited charge transport from low-temperature solution-processed oxide TFT in conjunction with high-*k* dielectrics is a challenging task.

As mentioned above, introducing a hybrid dielectric is a plausible solution; however, it typically fails to simultaneously achieve high-*k* and high dielectric strength. This is related to the low crosslinking efficiency of conventional crosslinking reagents such as silane coupling agents. For example, for the Al_2_O_3_–GPTMS–PMMA hybrid reported by Ramirez-Bon et al., a high molar ratio of the silane-based coupling agent GPTMS (molar ratio of Al_2_O_3_:GPTMS:PMMA = 1:7:0.5) limits the overall performance of the individual material. The excessive amount of the crosslinking agent decreases the dielectric constant due to the increase in organic content and induces defect states increasing the leakage current of the device^51^. Additionally, silanes are sensitive to moisture, consequently making crosslinking difficult under ambient conditions. Considering the detailed mechanism of the silane coupling agent, the dehydration reaction between an OH group (M–O–H) on the surface of the metal oxide and the OH group of the silane (C–Si–O–H) produces M–O–Si–C bonds, resulting in surface modification, changing the hydrophilic surface of the metal oxide to hydrophobic. Moreover, because of the high molar ratio of silane, a shell structure forms around the metal-oxide NPs, which reduces the intrinsic activity and limits the application of metal-oxide NPs^52^. Another disadvantage of this method is that silanes are highly reactive when appropriate acidic or basic conditions are met, which can result in product nonuniformity when mixed with polymers and metal oxides^53^. A more appropriate approach would be to use highly reactive nitrene chemistry with an azide coupling agent. However, as revealed in this study, simply mixing an azide coupling agent with organic and inorganic inclusions cannot guarantee the formation of covalent bonds between the inorganic oxide and organic polymers. Therefore, to realize genuine organic–inorganic hybrid dielectrics, we designed and synthesized acac-FPA, which enabled unprecedented high performance in conjunction with low-temperature solution-processed oxide semiconductors. Combined with temperature-dependent *I–V* analyses to determine the activation energy for transport, we demonstrated that a hybrid dielectric can also support the percolation-limited charge transport of oxide semiconductors.

To compare Hyb_2 with the state-of-the-art dielectric materials, In_2_O_3_/ZnO heterojunction TFT was fabricated with HfO_2_ dielectric layer deposited by atomic layer deposition and the resulting transfer characteristics are summarized in Supplementary Fig. 16. The extracted device parameters such as electron mobility (~22.51 cm^2^ V^−1^ s^−1^), on/off ratio (~10^5^), *V*_TH_ (0.53 V), and *SS* (280 mV cm^−1^) were lower in overall than the case of hybrid dielectric layer developed in this study.

In addition, to demonstrate whether the suggested azide-functionalized ligand can be applied to other types of oxide NPs, oxide TFT using PMMA–Al_2_O_3_ hybrid as a gate dielectric was fabricated with the same dielectric fabrication procedure using acac-FPA. The PMMA–Al_2_O_3_ hybrid dielectric layer showed a dielectric constant of 8.05 and dielectric strength of 4.06 MV cm^−1^ (Supplementary Fig. 17). The resulting In_2_O_3_/ZnO heterojunction TFTs with PMMA-Al_2_O_3_ as a gate dielectric also showed high performances overall as summarized in Supplementary Fig. 18a and Supplementary Table 5. The output curve of oxide TFTs with PMMA-Al_2_O_3_ as a gate dielectric also showed favorable linearity and effective saturation in the low *V*_DS_ and high *V*_DS_ regimes, respectively (Supplementary Fig. 18b). Bias-stress stability was also tested under both PBS and NBS conditions. In_2_O_3_/ZnO heterojunction TFT with PMMA-Al_2_O_3_ as a gate dielectric showed a low △*V*_TH_ (1.98 and −0.96 V under PBS and NBS conditions, respectively) against 43,200 s of bias-stress time (Supplementary Fig. 19 and Supplementary Table 6).

## Discussion

In summary, we demonstrated near-ideal organic–inorganic hybrid dielectric layers for high-performance solution-processed oxide transistors by achieving covalent networking between organic and inorganic inclusions. To guarantee crosslinking between the inorganic and organic phases, we synthesized acac-FPA, which can be both a chelating ligand of ZrO_2_ NPs and a photocrosslinker of ZrO_2_ NPs and PMMA. The experimentally and theoretically estimated porosity of the optimized hybrid dielectric was merely 1.4%, which could yield a significantly enhanced dielectric strength. Under the optimized conditions for the proposed hybrid dielectric layer, a high dielectric constant of ~14 and a high dielectric strength of over 4.0 MV cm^−1^ were achieved, and further analysis combined with contact angle and AFM analysis confirmed that this PMMA–ZrO_2_ hybrid dielectric layer was defect-free, with a superior dielectric-channel interface and a denser dielectric. This synergetic effect of the hybrid dielectric layer enabled the demonstration of exceptionally high-performance n-type oxide transistors comprising an In_2_O_3_/ZnO double layer as an active channel with electron mobility of over 50 cm^2^ V^−1^ s^−1^, on/off ratio of ~10^7^, and subthreshold swing of 108 mV dec^−1^. The bias-stress stability demonstrated that the proposed stable hybrid dielectric can effectively reduce the operational instability while retaining the TFT high performance. In addition, the temperature dependence of the TFTs, combined with carrier transport mechanism analysis, demonstrated that carrier transport is predominantly controlled by percolation. This study shows that the development of stable dielectric layers using cross-linkable ligands is an effective way to realize high-performance percolation mechanisms in solution-processed oxide TFTs.

## Methods

### Materials

All reagents were obtained from commercial suppliers and were used without further purification. Methyl pentafluorobenzoate, sodium azide, 4-(dimethylamino)pyridine (DMAP), and ethylene glycol were purchased from TCI. Copper (I) iodide, l-proline, acetylacetone, potassium carbonate, dimethyl sulfoxide, sodium hydroxide, hydrochloric acid, N,N’-dicyclohexylcarbodiimide (1.0 M in methylene chloride), Chloroform-d (CDCl_3_), acetone, methanol, anhydrous dichloromethane, PMMA, zirconium(IV) acetylacetonate (Zr(C_5_H_7_O_2_)_4_), aluminum acetylacetonate (Al(C_5_H_7_O_2_)_3_), indium nitrate hydrate (In(NO_3_)_3_·*x*H_2_O), zinc acetate dihydrate (Zn(CH_3_COO)_2_·2H_2_O), 2-methoxyethanol, DMF, and ethanolamine were purchased from Sigma Aldrich. MgSO_4_, n-hexane, ethyl acetate, and diethyl ether were purchased from Daejung Chemicals Korea.

### Synthesis of bis-FPA

Bis-FPA was synthesized following the procedure described in Supplementary Information. The synthesis scheme is shown in Supplementary Fig. 20.

### Synthesis of acac-FPA

#### Synthesis of 2-hydroxyethyl 4-azido-2,3,5,6-tetrafluorobenzoate

A mixture of 4-azido-2,3,5,6-tetrafluorobenzoic acid (1 g, 4.25 mmol), ethylene glycol (2.3 mL, 42.53 mmol), and anhydrous dichloromethane (25 mL) was stirred at room temperature followed by the addition of DMAP (51 mg, 0.42 mmol). After 30 min, the temperature was lowered to 0 °C and DCC (1 M in dichloromethane) (4.67 mL, 4.67 mmol) was added under continuous N_2_ purging. After 24 h, the reaction mixture was neutralized with water and extracted with dichloromethane, which was dried over MgSO_4_ followed by the removal of the solvent by evaporation at reduced pressure. The resulting crude product was purified by silica gel column chromatography using ethyl acetate/n-hexane as the eluent (2/5), yielding 2-hydroxyethyl 4-azido-2,3,5,6-tetrafluorobenzoate (yield 65%).

#### Synthesis of 3-(4-Benzoic acid)pentane-2,4-dione

3-(4-Benzoic acid)pentane-2,4-dione was synthesized in our laboratory according to a previously reported procedure^54^. A mixture of 4-iodobenzoic acid (1.1 g, 4.5 mmol), acetylacetone (1.4 mL, 13.5 mmol), K_2_CO_3_ (3.1 g, 22.5 mmol), CuI (0.25 g, 1.35 mmol), and l-proline (0.1 g, 0.9 mmol) in 15 mL of DMSO was stirred at room temperature for 5 min followed by heating at 90 °C for 24 h under continuous nitrogen purging. After 24 h, the solution was cooled and poured into a 1 M HCl solution to adjust the pH to neutral conditions and was then extracted with ethyl acetate. The organic layer was dried over MgSO_4_ and the solvent was removed under a vacuum. The crude oil was purified on a silica gel column using a mixture of hexane/ethyl acetate (1:1) as the eluent to afford 3-(4-benzoic acid)pentane-2,4-dione (yield 54%).

#### Synthesis of 2-((4-(1-acetyl-2-hydroxy-1-propen-1-yl)benzoyl)oxy)ethyl 4-azido-2,3,5,6-tetrafluorobenzoate (acac-FPA)

A mixture of 2-hydroxyethyl 4-azido-2,3,5,6-tetrafluorobenzoate (0.35 g, 1.25 mmol), 3-(4-benzoic acid)pentane-2,4-dione (0.25 g, 1.14 mmol), and anhydrous dichloromethane (25 mL) was stirred at room temperature followed by the addition of DMAP (13 mg, 0.11 mmol). After 30 min, the temperature was lowered to 0 °C, and DCC (1 M in dichloromethane) (1.25 mL, 1.25 mmol) was added under continuous N_2_ purging. After 24 h, the reaction mixture was neutralized with water, extracted with dichloromethane, and then dried over MgSO_4_ followed by the removal of the solvent by evaporation at reduced pressure. The resulting crude product was purified via silica gel column chromatography using ethyl acetate/n-hexane (1/5) as the eluent, yielding acac-FPA as a slightly yellow solid (340 mg, 56%).

^1^H NMR (400 MHz, CDCl_3_): δ = 1.90 (s, 6H), 1.81 (t, 2H), 1.81 (t, 2H), 7.29 (d, 2H), 8.09 (d, 2H) (Supplementary Fig. 21).

^13^C NMR (CDCl_3_): δ = 24.13, 62.31, 64.03, 114.47, 128.85, 130.22, 131.34, 142.29, 165.87, 190.57 (Supplementary Fig. 22).

^19^F NMR (CDCl_3_): δ = 138.39, 150.75 (Supplementary Fig. 23).

HRMS (ESI): Calculated for C_21_H_14_N_3_O_6_F_4_ [M–H]^−^: 480.0819, found: 480.0824.

### Precursor preparation

For PMMA-ZrO_2_ or PMMA-Al_2_O_3_ precursor solution, 0.1 g of PMMA and 0.1 M of Zr(C_5_H_7_O_2_)_4_ or Al(C_5_H_7_O_2_)_3_ were dissolved in 5 mL of DMF as a solvent and 30.18 µL of ethanolamine as a stabilizer. To crosslink the PMMA and oxide NPs, bis-FPA and acac-FPA were mixed with 5 wt% of PMMA and stirred at 60 °C for 3 h. For spin coating of In_2_O_3_, In(NO_3_)_3_·*x*H_2_O was dissolved in 2-methoxyethanol to achieve a 0.1 M and stirred at room temperature for 3 h before use. For spin coating of ZnO, 1.0 g of Zn(CH_3_COO)_2_·2H_2_O was dissolved in 10 mL of 2-methoxyethanol and 0.28 mL of ethanolamine and it was stirred at 60 °C for 3 h before use.

### Thin film fabrication

Highly doped silicon (Si^++^) was used as the substrate of TFT. Si wafers were cut to dimensions of 2 cm × 2 cm, and to remove organic residues from substrates, these were cleaned by piranha solution which is a mixture of sulfuric acid (H_2_SO_4_): 30% of hydrogen peroxide (H_2_O_2_) (7:3), followed by O_2_ plasma for 10 min. To cast the crosslinked PMMA–ZrO_2_ or PMMA–Al_2_O_3_ dielectric layers, the PMMA–ZrO_2_ or PMMA–Al_2_O_3_ sol was filtered by 0.2 µm PTFE filter and spin-coated onto pre-cleaned Si wafer at 2000 rpm for 50 s and the resulting films were exposed to 254 nm UV light from a 15 W lamp for 15 min to activate the crosslinking reaction at room temperature. Then, the substrates were annealed at 180 °C for 1 h. For the heterojunction active layer, the In_2_O_3_ precursor solution was first spin-coated at 3000 rpm for 30 s and subsequently annealed at 200 °C for 1 h. After cooling for enough time, the ZnO precursor solution was spin-coated at 4000 rpm for 30 s and subsequently annealed at 200 °C for 1 h. Finally, 100 nm-thick Al source and drain electrodes were deposited by thermal evaporation through a shadow mask with channels having a width of 300 μm and a length of 150 μm, as shown in Supplementary Fig. 24.

### Device characterization

The electrical characterizations of the transistors such as transfer curve, output curve, leakage current density, breakdown voltage, and bias-stress stability characteristics were performed at room temperature in a nitrogen glove box using a Keithley 2450 parameter analyzer. The electron mobility was evaluated in the saturation region using the following equation^50^:

10 $${\mu }_{{{{{{\rm{sat}}}}}}}=\frac{L}{W{C}_{1}}\frac{{\partial }^{2}{I}_{{{{{{\rm{D}}}}}},{{{{{\rm{sat}}}}}}}}{\partial {V}_{{{{{{\rm{G}}}}}}}^{2}},$$

where *L* and *W* are the length and width of the channel, respectively, *C*_1_ is the capacitance of the gate dielectric, *I*_D,sat_ is the saturation drain current, and *V*_G_ is the gate voltage.

^1^H NMR, ^13^C NMR, and ^19^F NMR spectra were recorded on a Bruker Avance-400 spectrometer using TMS as internal standard. Chemical shifts are reported in parts per million (ppm). CDCl_3_ was used as the NMR solvent in all cases. High-resolution mass spectra were obtained with an Ion Mobility Q-TOF (Agilent, G6560A). AFM (Park Systems, XE150) was used to measure the surface morphology and roughness. SEM (Hitachi, S-4800) was used to obtain the surface feature, distribution, and thickness of the film. TEM (JEOL, JEM-2200FS) was used to analyze the particle shape of the hybrid dielectric layer. XPS (Thermo Scientific, ESCALAB 250) and PLS-II 4D beamline at the Pohang Accelerator Laboratory (PAL) in Korea were used to analyze the chemical composition of the hybrid dielectric layer. A Bruker vertex 70v was used to obtain the FT-IR transmittance peaks. A Thermo Scientific Evolution 220 UV–vis spectrophotometer was used to obtain the UV–vis absorption spectra. Contact angles were measured using an Ossila L2004A1 goniometer. Temperature-dependent transfer characteristics were measured using a Janis ST-500-5CX-1CXKEL and Keithley 2450 parameter analyzer.

## Supplementary information

Supplementary Information

## Data Availability

The data that support the findings of this study within this article and its Supplementary Information files are provided with this paper. Source data are provided with this paper.

## Source data

Source Data
