# Supplementary material for: Azide-functionalized ligand enabling organic–inorganic hybrid dielectric for high-performance solution-processed oxide transistors
Source: Nat Commun. 2022 Nov 17;13:7021. doi: 10.1038/s41467-022-34772-x (PMC9671905; doi:10.1038/s41467-022-34772-x)
Supplement: Supplementary file 1 — Supplementary Information [file 41467_2022_34772_MOESM1_ESM.pdf]

Supplementary Information

Azide-functionalized Ligand Enabling Organic–Inorganic Hybrid Dielectric for High-Performance Solution-Processed Oxide Transistors

Juhyeok Lee[1], Syed Zahid Hassan[1], Sangjun Lee[1], Hye Ryun Sim[1], Dae Sung Chung[1]*

[1]Department of Chemical Engineering, Pohang University of Science and Technology (POSTECH), Pohang 37673, Republic of Korea.

*Correspondence: dchung@postech.ac.kr

Synthesis of bis-FPA

(1) Synthesis of methyl 4-azido-2,3,5,6-tetrafluorobenzoate

The methyl pentafluorobenzoate (5 g, 22.11 mmol) was dissolved in a mixture of acetone (60 mL) and water (30 mL) under stirring condition followed by slow addition of sodium azide (2.16 g, 33.17 mmol) to the reaction flask. After refluxing at 60 °C for 12 h, the reaction mixture was cooled to room temperature, extracted with diethyl ether and concentrated under reduced pressure. The completion of reaction was confirmed by checking TLC. Crude product was used for next step without characterization.

(2) Synthesis of 4-azido-2,3,5,6-tetrafluorobenzoic acid

To the solution of 4-azido-2,3,5,6-tetrafluorobenzoate (2 g, 8.03 mmol) in methanol (50 mL) and water (5 mL), aqueous solution of NaOH (20% w/w, 12.04 mmol, 1.5 equivalents) were added in the reaction flask. Solution was stirred overnight at room temperature followed by neutralization with HCl (2 M) by adjusting pH to ~1. Reaction mixture was extracted with diethyl ether and concentrated under reduced pressure. The completion of reaction was confirmed by checking TLC which yielded 4-azido-2,3,5,6-tetrafluorobenzoic acid. Crude product was used for next step without characterization.

(3) Synthesis of ethane-1,2-diyl bis(4-azido-2,3,5,6-tetrafluorobenzoate), bis-FPA

A mixture of 4-azido-2,3,5,6-tetrafluorobenzoic acid (1 g, 4.25 mmol) and ethylene glycol (107 μL, 1.91 mmol) were dissolved in anhydrous dichloromethane (25 mL) under stirring at room temperature followed by addition of DMAP (51 mg, 0.42 mmol). After 30 min, temperature was lowered to 0 °C, and DCC (1 M in dichloromethane) (4.67 mL, 4.67 mmol) was added under N_2

atmosphere. After 12 h, water was added to the reaction mixture and extracted with dichloromethane and concentrated under reduced pressure. The resulting crude product was purified by silica gel column chromatography using an eluent of ethyl acetate/n-hexane (1/5), which yielded the bis-FPA as a colorless solid (560 mg, 58%). ^{1}H NMR (400 MHz, $CDCl_3$): δ = 4.71 (s, 4H). ^{19}F NMR (376.5 MHz, $CDCl_3$): δ = -138.16 -138.25 (m), -150.69 -150.80 (m). ^{1}H NMR and ^{19}F NMR of synthesized bis-FPA matches with previously reported value[1].

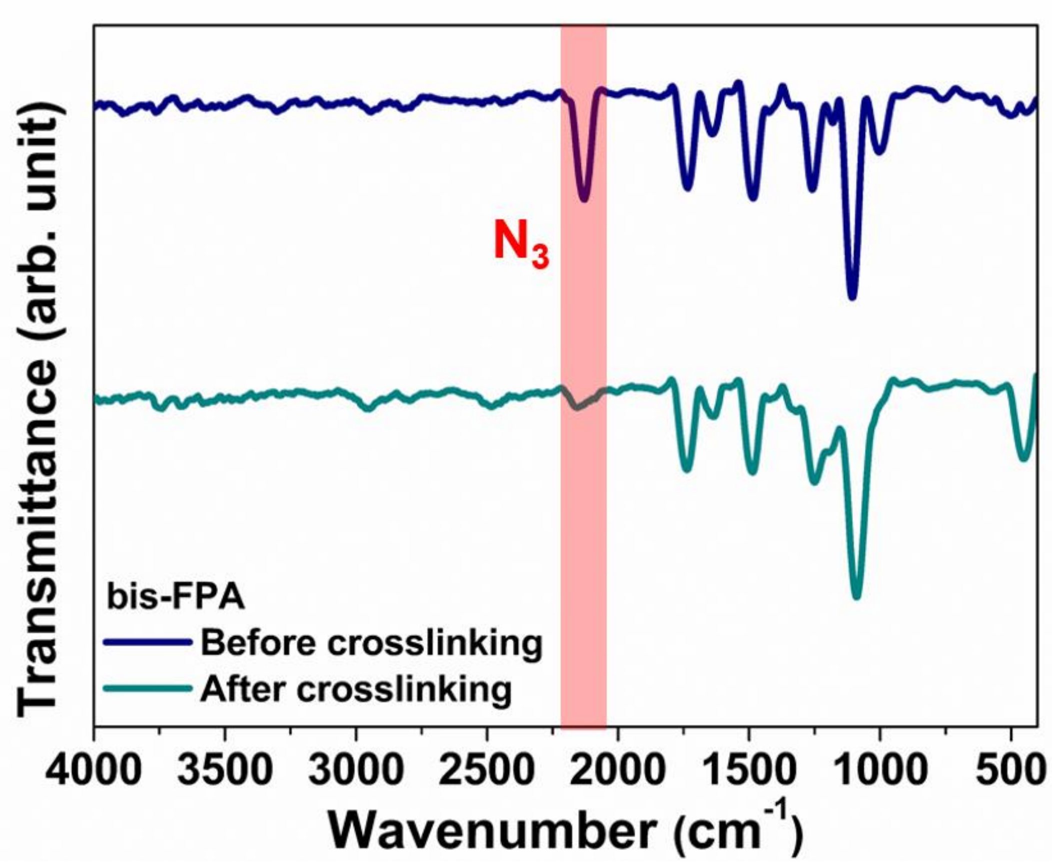

Supplementary Fig. 1. FT-IR spectra of bis-FPA films before and after being crosslinked. Red shade represents the presence of azide groups via the asymmetric stretching band ν_{as} (N_3).

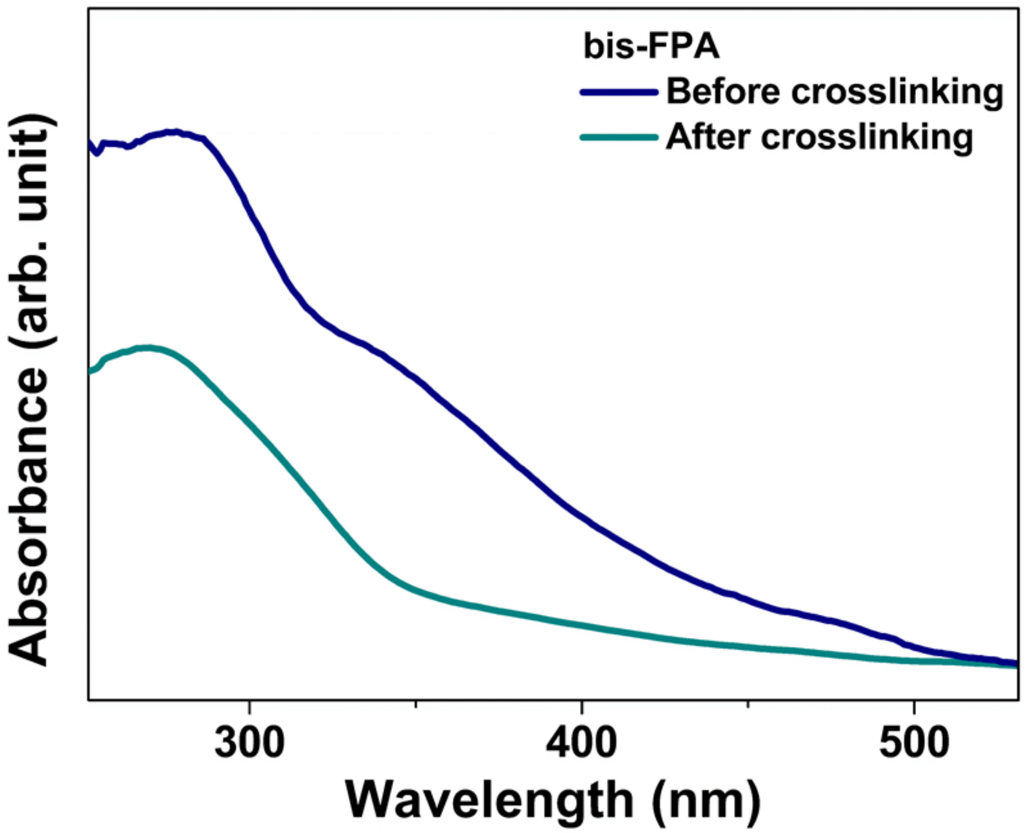

Supplementary Fig. 2. UV-visible absorption spectra of bis-FPA films before and after being crosslinked. The UV-vis absorption spectra of bis-FPA shows characteristic peaks of the FPA moiety in the range of 250–320 nm which subsequently reduce in intensity after UV light irradiation.

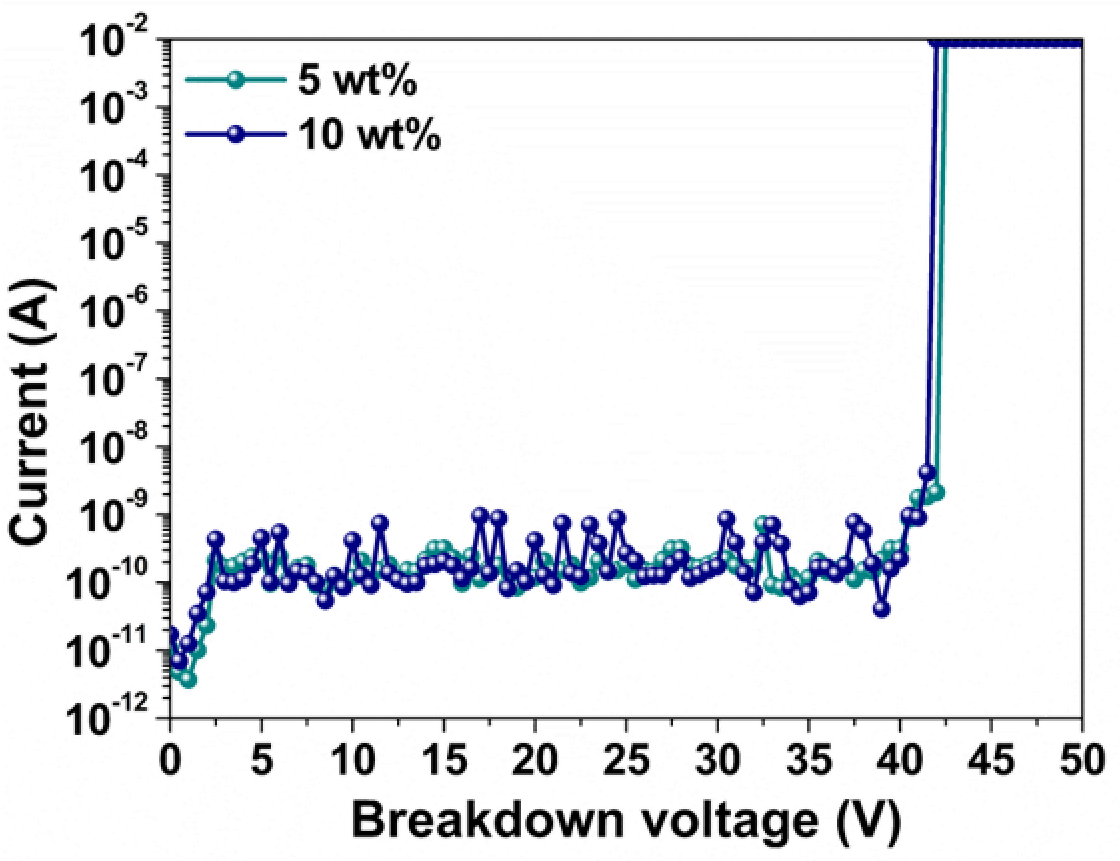

Supplementary Fig. 3. Breakdown voltage of PMMA-ZrO_2 dielectric with 5 wt% and 10 wt% of bis-FPA. PMMA/ZrO_2/bis-FPA with 10 wt% of bis-FPA gave very similar characteristics compared to Hyb_1 with 5 wt% of bis-FPA, in terms of breakdown voltage.

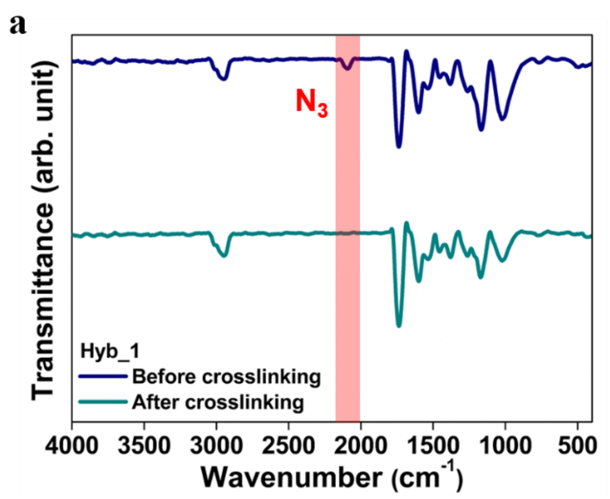

Supplementary Fig. 4. FT-IR spectra of Hyb_1 and Hyb_2 films before and after being crosslinked. The asymmetric stretching at 2093 and 2094 in the FT-IR spectra (red shades) of the **a** Hyb_1 and **b** Hyb_2 hybrids, respectively, indicate the presence of azide, which diminishes after UV light irradiation.

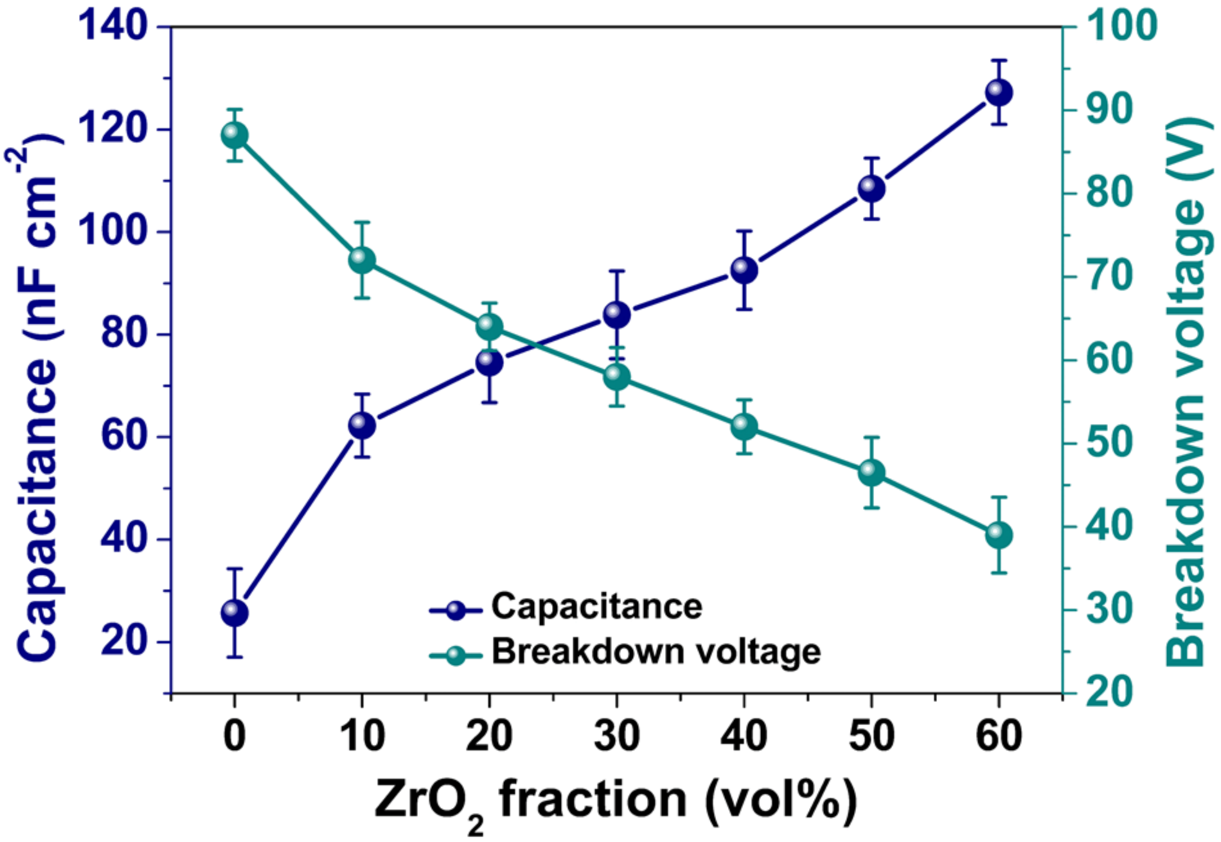

Supplementary Fig. 5. Capacitance and breakdown voltage as a function of ZrO_2 fractions. The areal capacitance values measured at 1 kHz and the breakdown voltages evidently increase and decrease, respectively, with increasing ZrO_2 NP loading ratios. Error bars represent standard deviation from 10 devices.

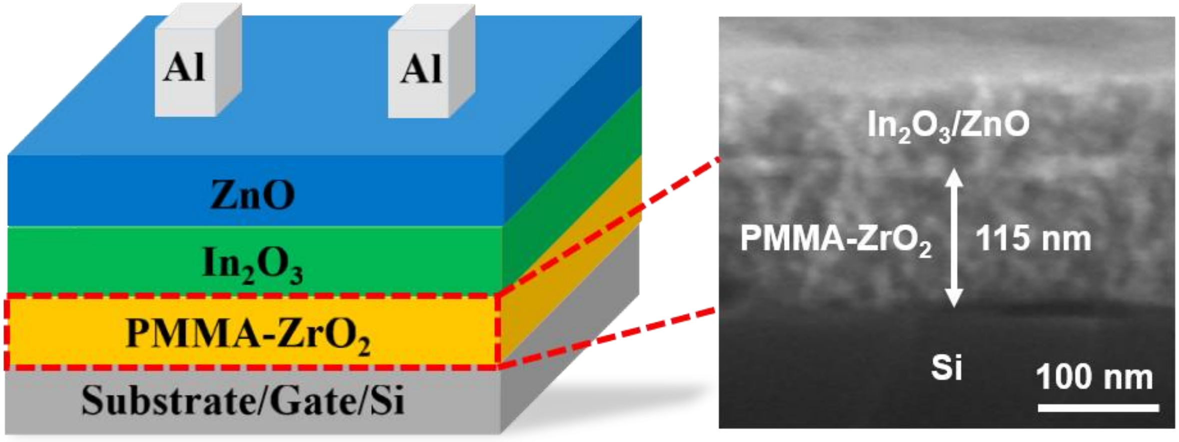

Supplementary Fig. 6. Schematic of the TFT structure and SEM cross-section image of the device. The thickness of the dielectric layer of PMMA-ZrO_2 is 115 nm, and the thickness of the active layer of In_2O_3/ZnO is 25 nm/25 nm.

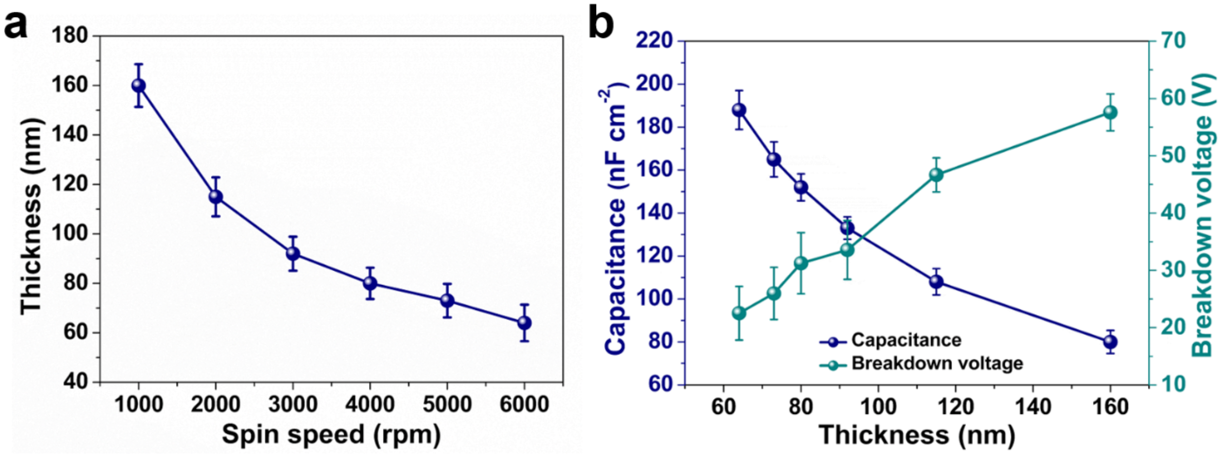

Supplementary Fig. 7. Characterizations of hybrid dielectric layer for thickness. a Thickness as a function of spin speed. Error bars represent standard deviation from 10 devices. **b** Capacitance and breakdown voltage as a function of thickness. Error bars represent standard deviation from 10 devices.

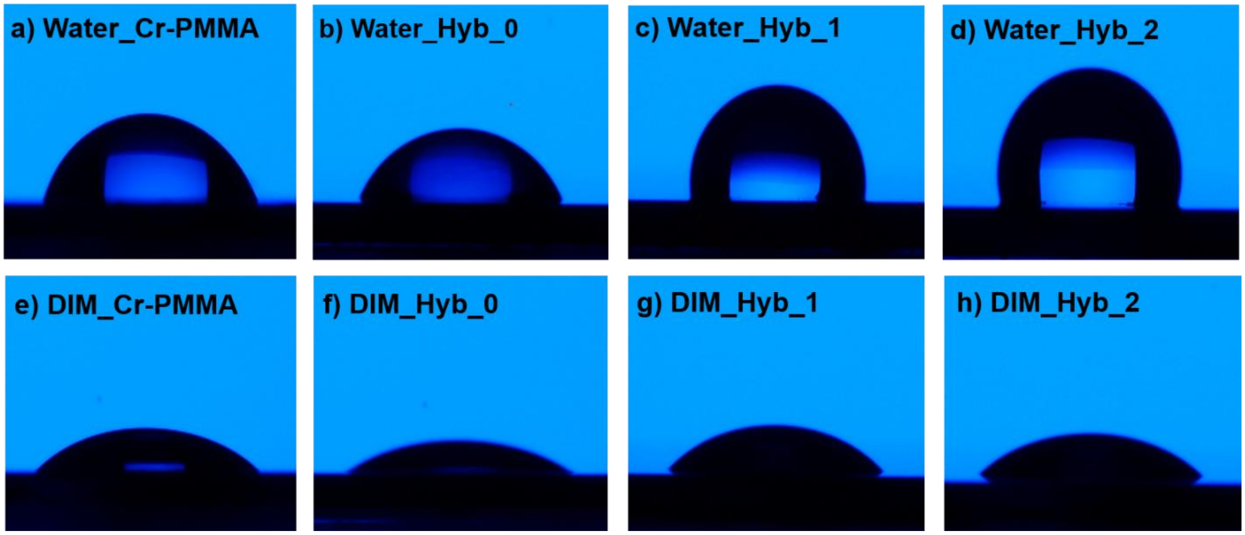

Supplementary Fig. 8. Water and diiodomethane contact angle on various surfaces. Contact angle images of **a-d** water and **e-h** DIM droplets on the surface of **a,e** crosslinked-PMMA, **b,f** Hyb_0, **c,g** Hyb_1, and **d,h** Hyb_2.

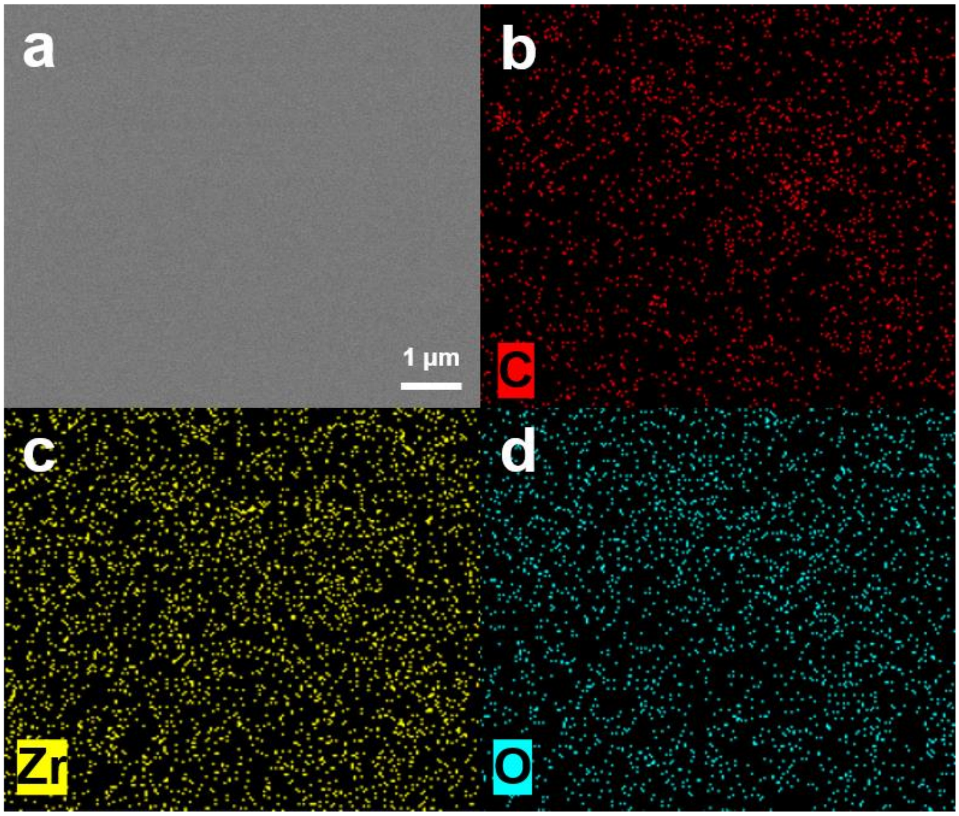

Supplementary Fig. 9. SEM image of Hyb_2 and cross-sectional EDS mapping, indicating the homogeneous element distribution. a Top-view SEM image of Hyb_2. Elemental distribution of EDS mapping for Hyb_2: **b** C element; **c** Zr element; and **d** O element.

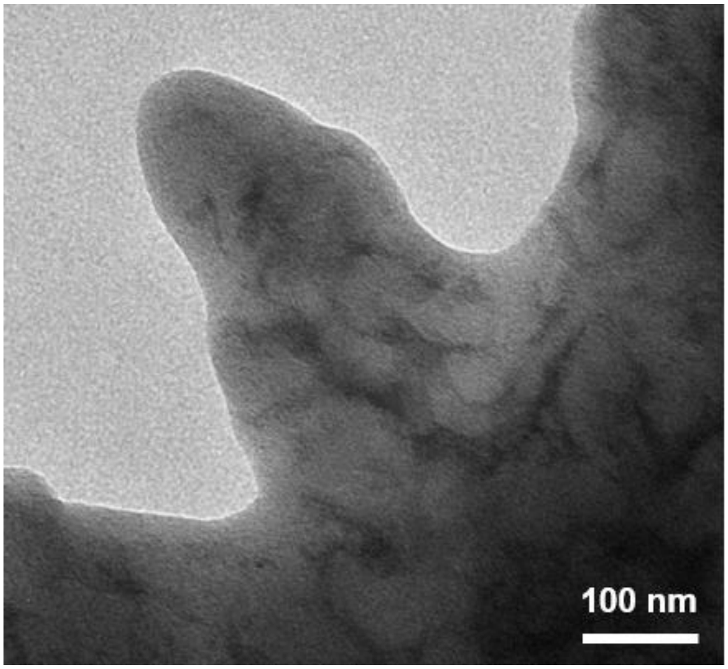

Supplementary Fig. 10. TEM image of Hyb_2. It shows that ZrO_2 clusters are well distributed within the matrix PMMA over the entire dielectric surface with cluster size less than 100 nm.

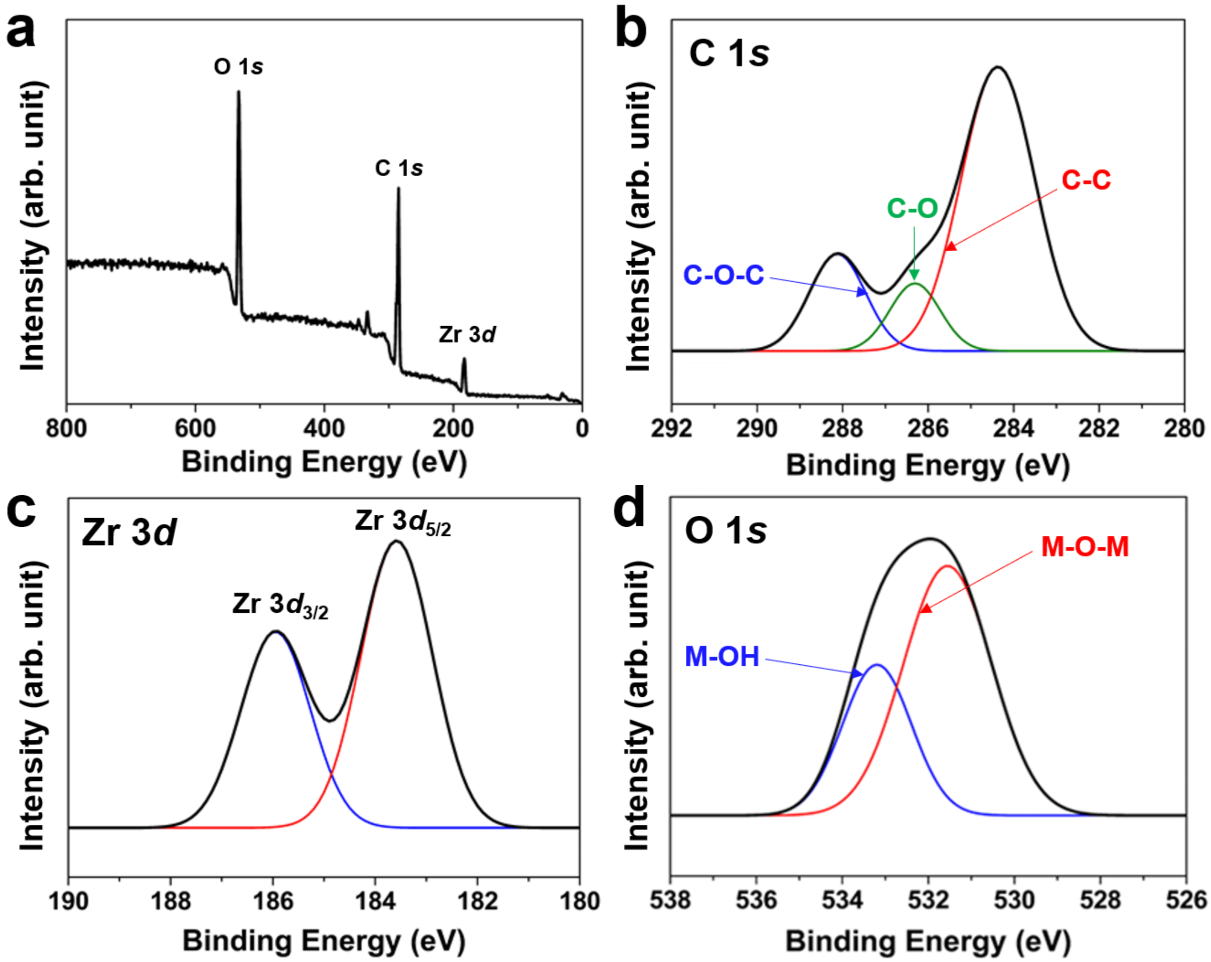

Supplementary Fig. 11. XPS results of Hyb_2. a XPS survey scan of Hyb_2. High resolution XPS spectra of **b** C 1*s* feature, **c** Zr 3*d* feature, and **d** O 1*s* feature.

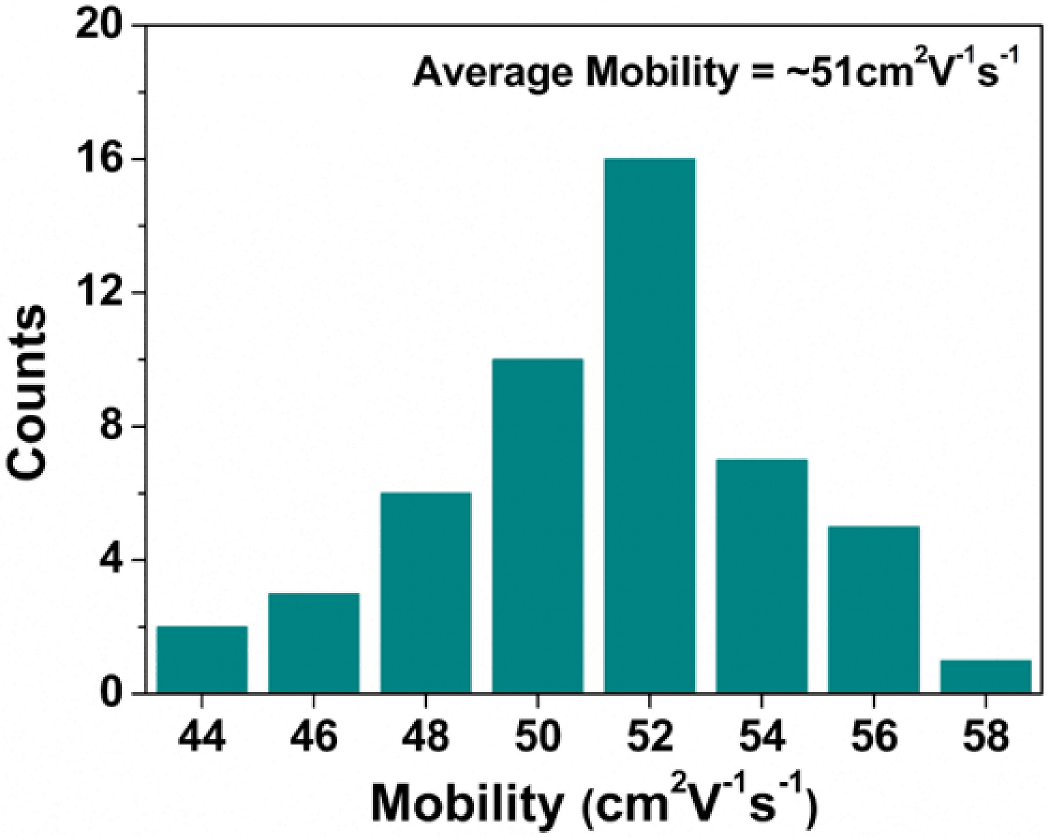

Supplementary Fig. 12. Mobility statistics of In_2O_3/ZnO heterojunction TFTs with Hyb_2 as a gate dielectric. It showed high device reproducibility, with an average electron mobility of ~51 cm^2 V^{-1} s^{-1} calculated from 50 independently fabricated devices.

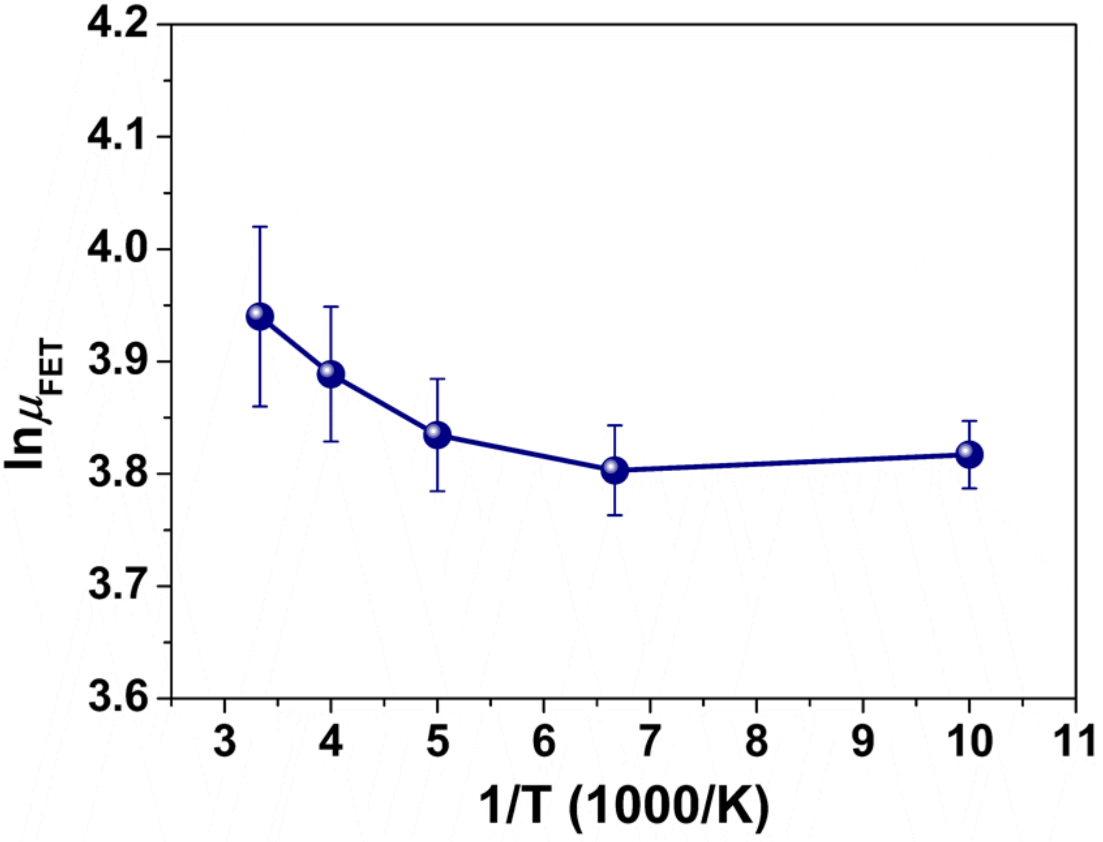

Supplementary Fig. 13. Temperature dependence of electron mobilities. The activation energy, E_a, determined from the 200 K ≤ T ≤ 300 K region was ~5.4 meV, indicating a practically negligible trap state density. Error bars represent standard deviation from 10 devices.

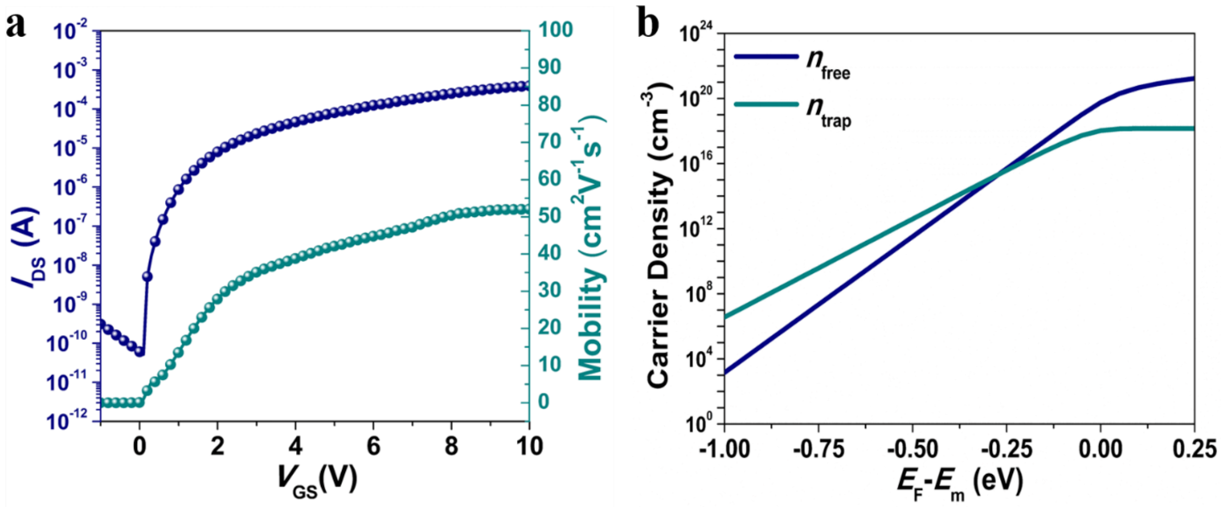

Supplementary Fig. 14. Carrier density characteristics of In_2O_3/ZnO heterojunction TFT with Hyb_2 as a gate dielectric. a Drain-source current (I_{DS}) and electron mobility as a function of gate-source voltage. (V_{GS}) **b** Carrier densities (n_{free} and n_{trap}) as a function of Fermi energy level (E_F) from the conduction band edge. (E_m)

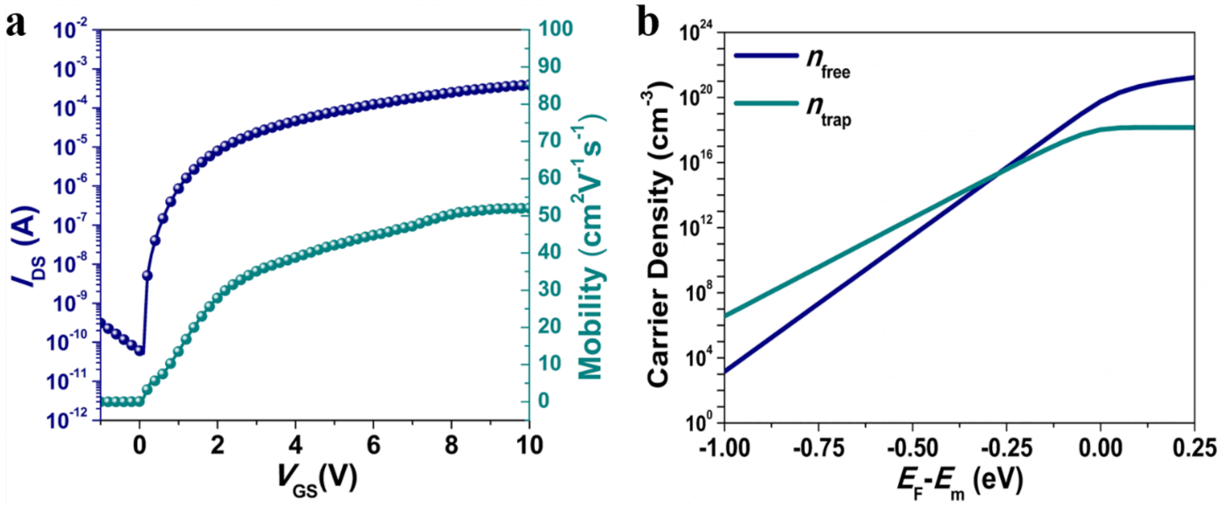

Supplementary Fig. 15. Carrier density characteristics of In_2O_3/ZnO heterojunction TFT with SiO_2 as a gate dielectric. a Drain-source current (I_{DS}) and electron mobility as a function of gate-source voltage. (V_{GS}) **b** Carrier densities (n_{free} and n_{trap}) as a function of Fermi energy level (E_F) from the conduction band edge. (E_m)

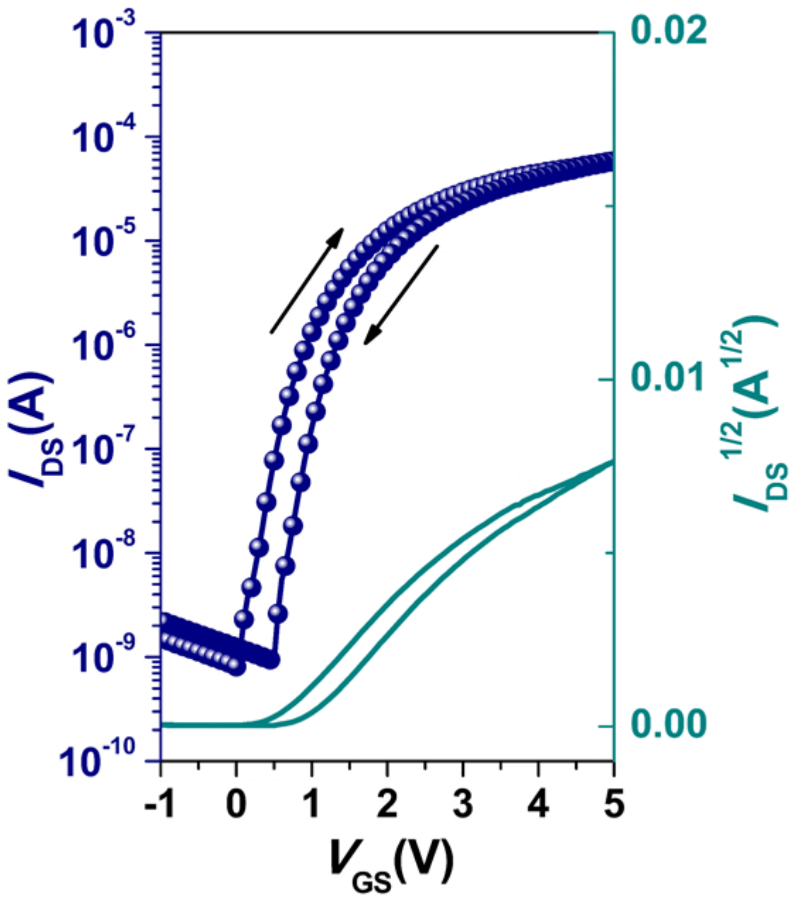

Supplementary Fig. 16. Representative transfer characteristics of In_2O_3/ZnO heterojunction TFT with HfO_2 gate dielectric layer grown by atomic layer deposition. (V_{DS} = 5 V)

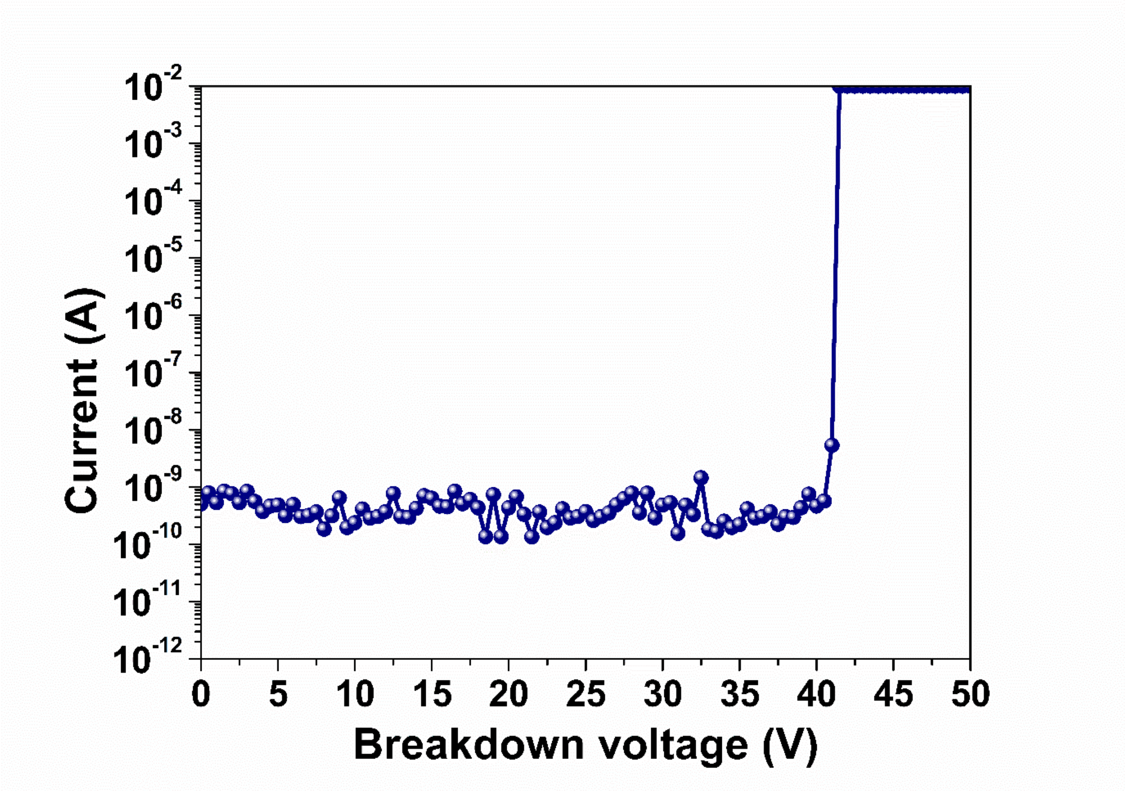

Supplementary Fig. 17. Breakdown voltage of PMMA-Al_2O_3 dielectric.

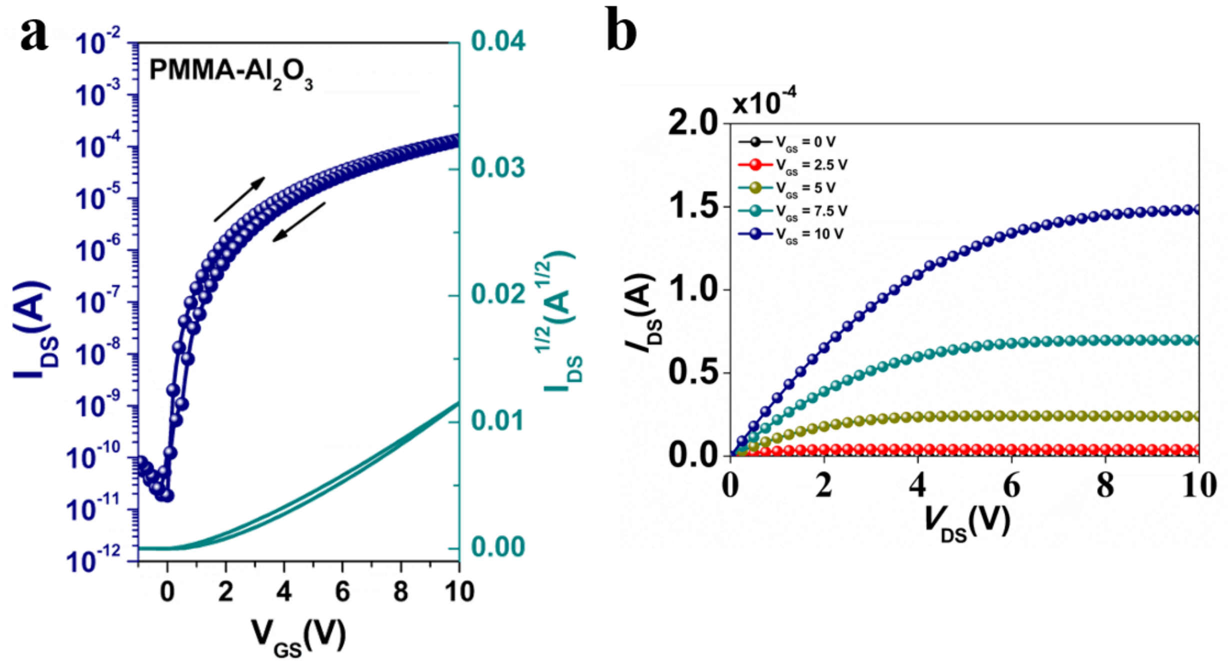

Supplementary Fig. 18. Current-voltage characteristics of In_2O_3/ZnO heterojunction TFT with PMMA-Al_2O_3 as a gate dielectric. a Transfer characteristics of the TFT with PMMA-Al_2O_3 as a gate dielectric, measured at V_D = 10 V. **b** Output characteristics of the TFT with PMMA-Al_2O_3 as a gate dielectric. The gate voltage was increasingly varied between 0 V and 10 V in steps of 2.5 V.

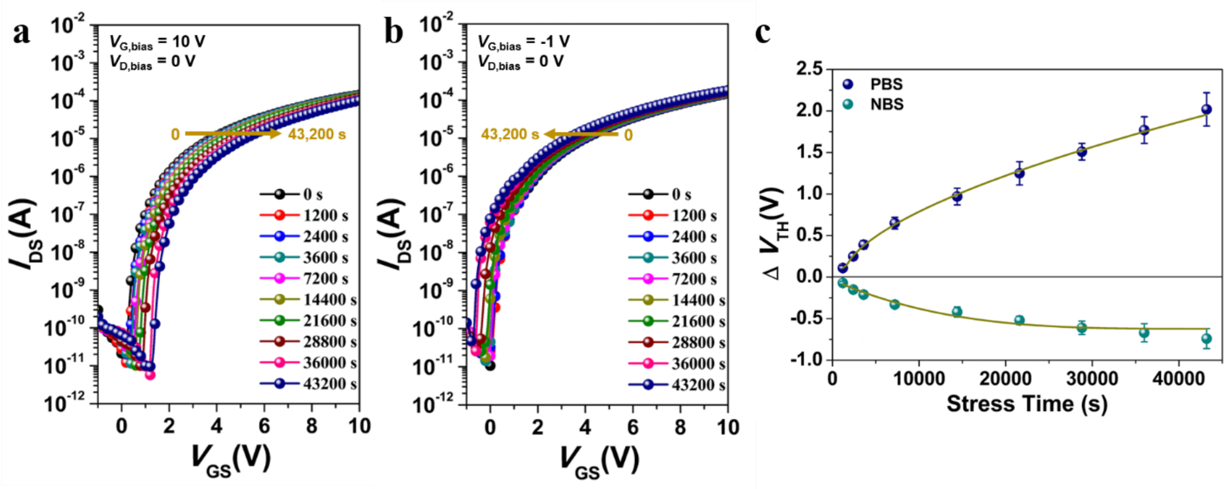

Supplementary Fig. 19. Operational bias-stress stability of In_2O_3/ZnO heterojunction TFTs with PMMA-Al_2O_3 as a gate dielectric. a Transfer characteristics under PBS. ($V_{G,bias}$ = 10 V, $V_{D,bias}$ = 0 V) **b** Transfer characteristics under NBS. ($V_{G,bias}$ = -1 V, $V_{D,bias}$ = 0 V) The bias stress time was varied between 0 s and 43200 s. **c** Time dependence of $\triangle V_{TH}$ as a function of stress time under PBS and NBS conditions. Error bars represent standard deviation from 10 devices.

Synthetic scheme

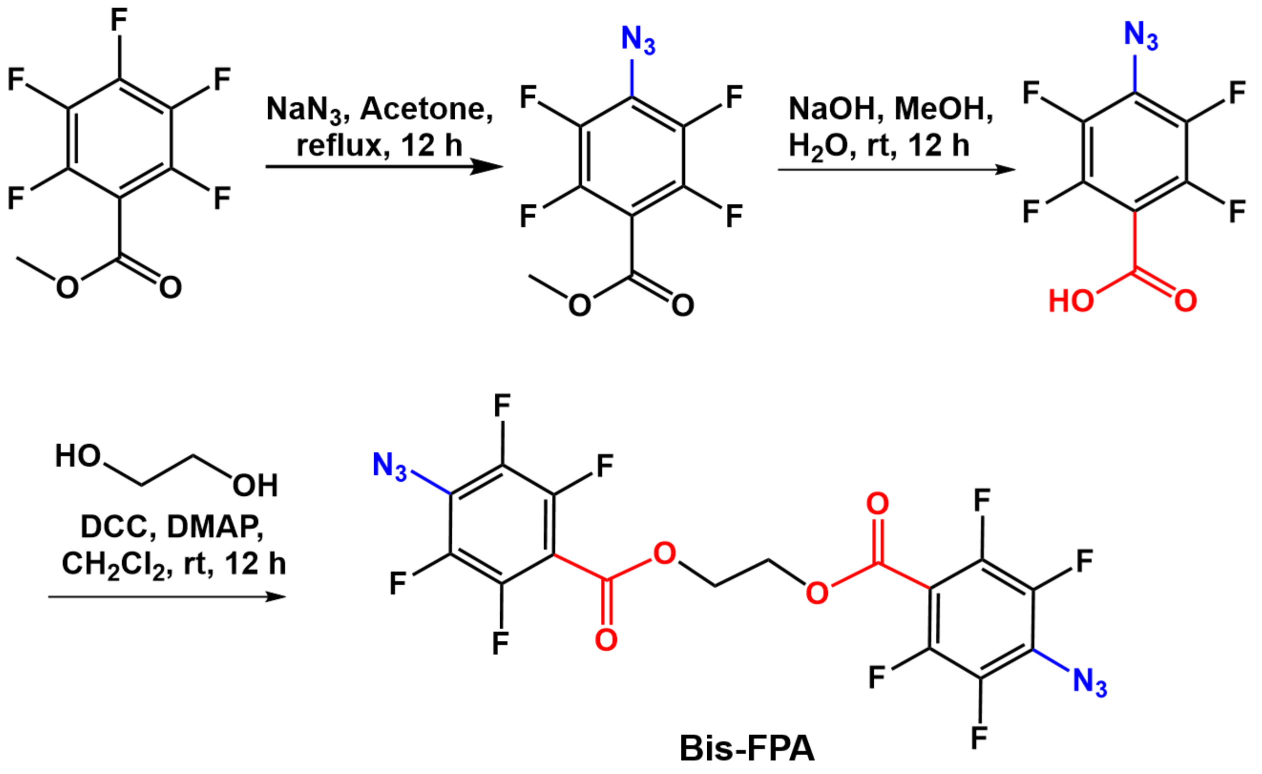

Supplementary Fig. 20. Synthetic procedure of ethane-1,2-diyl bis(4-azido-2,3,5,6-tetrafluorobenzoate).

^{1}H NMR

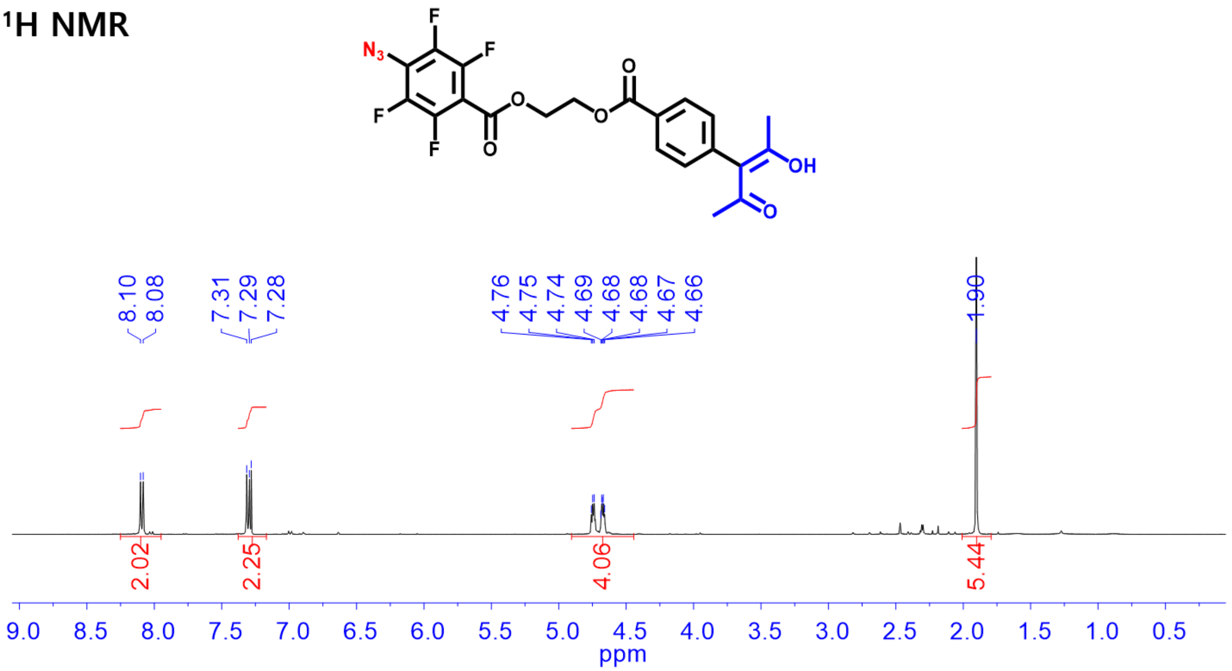

Supplementary Fig. 21. ^{1}H NMR data of 2-((4-(1-acetyl-2-hydroxy-1-propen-1-yl)benzoyl)oxy)ethyl 4-azido-2,3,5,6-tetrafluorobenzoate.

^{13}C NMR

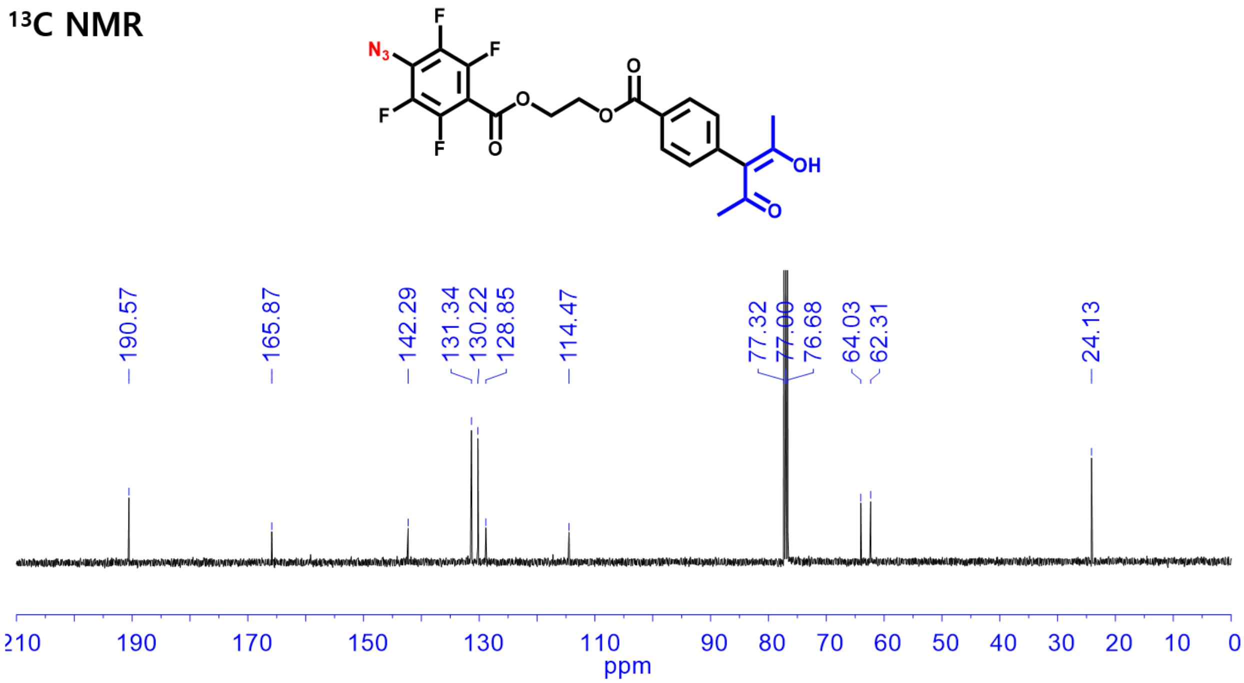

Supplementary Fig. 22. ^{13}C NMR data of 2-((4-(1-acetyl-2-hydroxy-1-propen-1-yl)benzoyl)oxy)ethyl 4-azido-2,3,5,6-tetrafluorobenzoate.

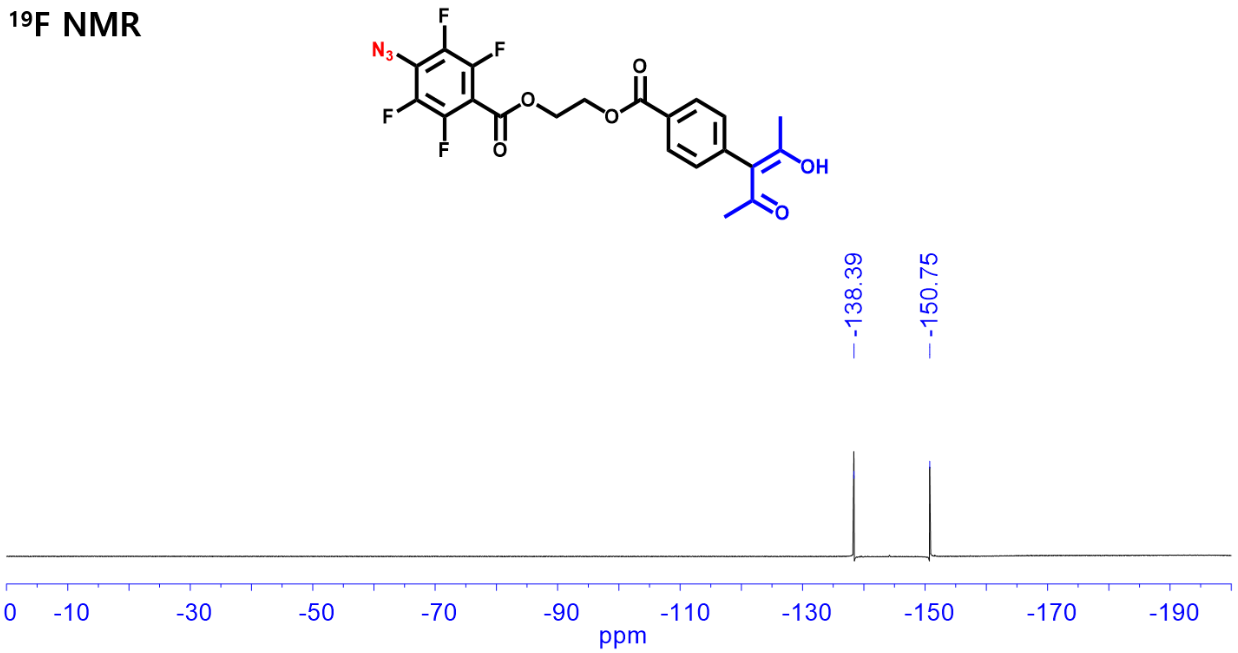

Supplementary Fig. 23. ^{19}F NMR data of 2-((4-(1-acetyl-2-hydroxy-1-propen-1-yl)benzoyl)oxy)ethyl 4-azido-2,3,5,6-tetrafluorobenzoate.

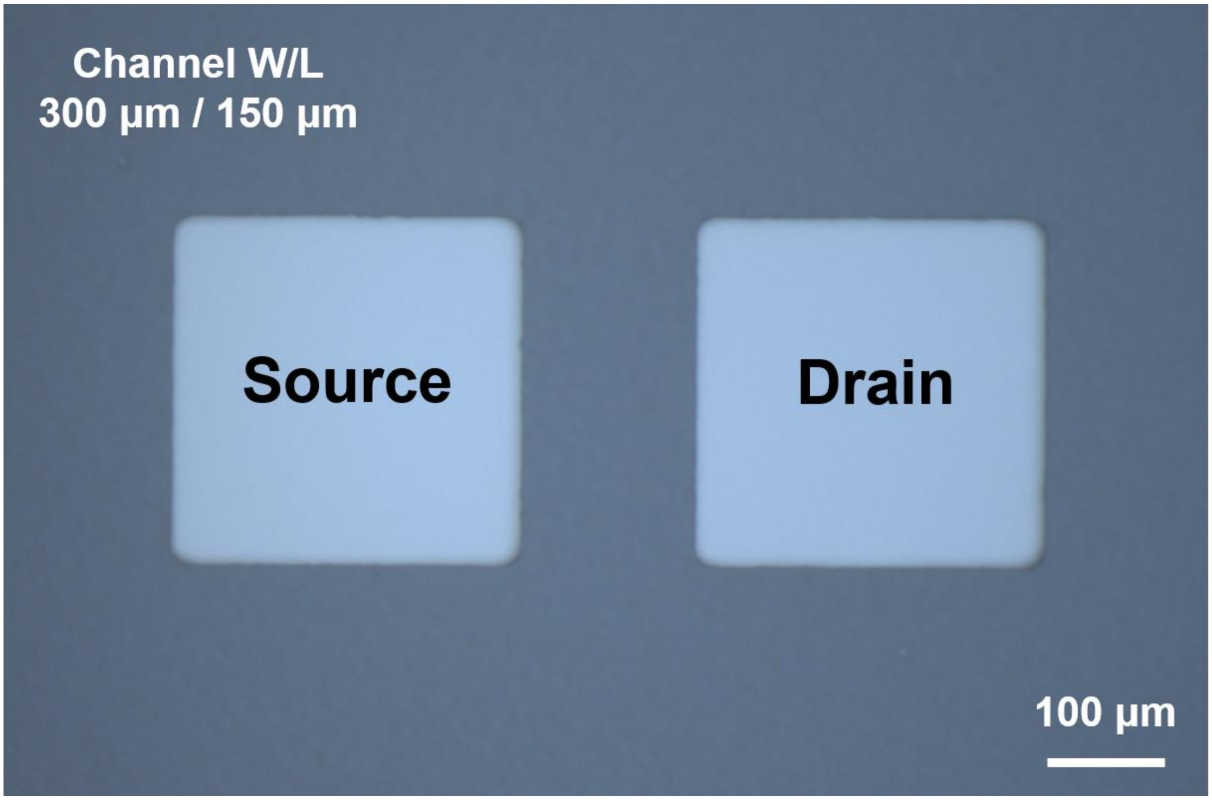

Supplementary Fig. 24. Optical microscope image of TFT fabricated on Si (Gate) substrate. TFT channel width/length is 300 µm/150 µm.

Supplementary Table 1. Summary of contact angles measured on various surfaces (crosslinked-PMMA, Hyb_0, Hyb_1, and Hyb_2) and surface energy values calculated on basis of them.

	θ_{water} (°)	θ_{DIM} (°)	γ (mN m^{-1})
Crosslinked-PMMA	70.92	37.54	47.51
Hyb_0	53.87	28.21	58.77
Hyb_1	92.69	41.29	39.55
Hyb_2	97.89	42.62	38.39

Supplementary Table 2. Comparison of dielectric properties and electrical characteristics for various n-MOS with different organic-inorganic hybrid gate dielectric materials.

**Dielectric material**	**Channel material**	***k***	**Mobility ($cm^2V^{-1}s^{-1}$)**	I_{on}/I_{off}	V_{TH} **(V)**	***SS*** **(V dec^{-1})**	**Ref.**
PVP-*co*-PMMA-PMF-HfO_x	IGTO	8.84	25.9	~10^7	-0.2	7	2
PVP-*co*-PMMA-PMF-ZrO_2	IZO	5.6	28.4	~10^7	-2.0	0.7	3
PMMA-GPTMS-Al_2O_3	ZnO	6.2	4.5	~10^7	0.7	-	4
PMMA-GPTMS-$ZrHfO_2$	ZnO, IGZO	9.4	12.8, 2.45	~10^3, ~10^7	2.5, 1.2	3, 0.68	5
PMMA-TMSPM-ZrO_2	ZnO	6.8	0.48	~10^5	3.3	0.47	6
PMMA-GPTMS-HfO_2	ZnO	11.3	15.5	~10^6	0.5	0.37	7
PVP/Al_2O_3	SIZO	-	8.84	~10^5	-1.5	1.09	8
PVP/ZrO_2:B	In_2O_3	8.5	0.44	~10^5	1.7	-	9
PVP/Al_2O_3	IGZO	-	8.39	~10^5	7.03	0.68	10
PVP-PMF-Al_2O_3	IGZO	6.1	5.13	~10^5	2.3	0.84	11
PVP-PMF-HfO_2	In_2O_3	6.5	2.6	~10^5	0.1	0.33	12
PVA-GO	IGZO	-	42	~10^7	0.79	0.106	13
GPTMS-HfO_2	IGZO	11.4	4.74	~10^4	0.3	-	14
PMMA-ZrO_2	In_2O_3/ZnO	14.0	51.19	~10^7	0.46	0.108	This work

Supplementary Table 3. Comparison of bias-stress stability characteristics for various n-MOS with different gate dielectric materials.

Dielectric material	Channel material	Stress time (s)	Gate stress (V)	τ (s)	β	Ref.
SiO_2	In_2O_3/ZnO	86,400	30	2.55×10^8	0.40	15
SiO_2	$In_2O_3/ZnO:Al$	86,400	30	6.03×10^3	1	
SiO_2	In_2O_3	13,200	10	3.64×10^4	0.53	16
SiO_2	IGZO	10,000	15	2.00×10^4	0.42	17
SiO_2	In_2O_3	32,400	40	2.34×10^5	0.51	18
SiO_2	ZnO	32,400	40	2.32×10^5	0.50	
SiO_2	IGZO	7,000	30	1.30×10^6	0.46	19
Al_2O_3	IGZO	10,000	5	-	-	20
ZrO_2	IZO	1,000	20	-	-	21
$AlZrO_x$	SWCNT	1,800	-3	2.63×10^3	-	22
HfO_y/AlO_x	ZTO	3,600	3	1.13×10^7	0.20	23
AlO_x/HfO_y	ZTO	3,600	3	0.65×10^3	0.44	
ZrO_2/HfO_2	IGZO	3,600	5	8.31×10^6	0.79	24
PMMA-ZrO_2	In_2O_3/ZnO	43,200	10	4.06×10^5	0.64	This work

Supplementary Table 4. Electrical characteristics of In_2O_3/ZnO heterojunction TFTs with Hyb_2 as a gate dielectric before/after bias-stress. Summary of characteristics of In_2O_3/ZnO heterojunction TFTs measured before and after 12 h of continuous bias-stress under PBS and NBS conditions.

Dielectric layer	Bias-stress conditions	Bias-stress time (s)	Mobility ($cm^2 V^{-1} s^{-1}$)	I_{on}/I_{off}	V_{TH} (V)	SS (mV dec^{-1})	D_{tr} ($eV^{-1} cm^{-2}$)
PMMA-ZrO_2	V_G = 10 V, V_D = 0 V	0	51.21	~10^7	0.46	106	5.19×10^{11}
		43200	48.22	~10^7	2.31	110	5.65×10^{11}
	V_G = -1 V, V_D = 0 V	0	51.90	~10^7	0.54	118	6.55×10^{11}
		43200	54.57	~10^7	0.06	117	6.43×10^{11}

Supplementary Table 5. Electrical characteristics of In_2O_3/ZnO heterojunction TFT with PMMA-Al_2O_3 as a gate dielectric.

**Dielectric layer**	**Capacitance (nF cm^{-2})**	**Active layer**	**Mobility (cm^{2}V^{-1}s^{-1})**	I_{on}/I_{off}	V_{TH} **(V)**	***SS*** **(mV dec^{-1})**	D_{tr} **(eV^{-1} cm^{-2})**
PMMA-Al_2O_3	64	In_2O_3 /ZnO	38.10±3.53	~10^7	0.62	129	4.61x10^{11}

Supplementary Table 6. Electrical characteristics of In_2O_3/ZnO heterojunction TFTs with PMMA-Al_2O_3 as a gate dielectric before/after bias-stress. Summary of characteristics of In_2O_3/ZnO heterojunction TFTs measured before and after 12 h of continuous bias-stress under PBS and NBS conditions.

Dielectric layer	Bias-stress conditions	Bias-stress time (s)	Mobility ($cm^2V^{-1}s^{-1}$)	I_{on}/I_{off}	V_{TH} (V)	*SS* (mV dec^{-1})	D_{tr} (eV^{-1} cm^{-2})
PMMA-Al_2O_3	V_G = 10 V<br>V_D = 0 V	0	38.62	~10^7	0.64	133	$4.88x10^{11}$
		43200	36.05	~10^7	2.62	158	$6.54x10^{11}$
	V_G = -1 V<br>V_D = 0 V	0	38.51	~10^7	0.65	130	$4.68x10^{11}$
		43200	42.34	~10^7	-0.31	127	$4.48x10^{11}$

Supplementary References

[1] Kim, J. *et al.* Synergetic contribution of fluorinated azide for high EQE and operational stability of top-illuminated, semitransparent, photomultiplication-type organic photodiodes. *Mater. Horiz.* **8**, 3141-3148 (2021).

[2] Hur, J. S., Kim, J. O., Kim, H. A. & Jeong, J. K. Stretchable Polymer Gate Dielectric by Ultraviolet-Assisted Hafnium Oxide Doping at Low Temperature for High-Performance Indium Gallium Tin Oxide Transistors. *ACS Appl. Mater. Interfaces* **11**, 21675-21685 (2019).

[3] Son, B.-G., Je, S. Y., Kim, H. J. & Jeong, J. K. Modification of a polymer gate insulator by zirconium oxide doping for low temperature, high performance indium zinc oxide transistors. *RSC Adv.* **4**, 45742-45748 (2014).

[4] Meza-Arroyo, J., Syamala Rao, M. G., Mejia, I., Quevedo- López, M. A. & Ramírez-Bon, R. Low temperature processing of Al_2O_3-GPTMS-PMMA hybrid films with applications to high-performance ZnO thin-film transistors. *Appl. Surf. Sci.* **467-468**, 456-461 (2019).

[5] Syamala Rao, M. G. *et al.* Low-temperature sol-gel $ZrHfO_2$-PMMA hybrid dielectric thin-films for metal oxide TFTs. *J. Non-Cryst. Solids* **502**, 152-158 (2018).

[6] Alvarado-Beltran, C. G., Almaral-Sanchez, J. L., Mejia, I., Quevedo-Lopez, M. A. & Ramirez-Bon, R. Sol-Gel PMMA-ZrO_2 Hybrid Layers as Gate Dielectric for Low-Temperature ZnO-Based Thin-Film Transistors. *ACS Omega* **2**, 6968-6974 (2017).

[7] Mullapudi, G. S. R., Velazquez-Nevarez, G. A., Avila-Avendano, C., Torres-Ochoa, J. A., Quevedo-López, M. A. & Ramírez-Bon, R. Low-Temperature Deposition of Inorganic–Organic

HfO_2–PMMA Hybrid Gate Dielectric Layers for High-Mobility ZnO Thin-Film Transistors. *ACS Appl. Electron. Mater.* **1**, 1003-1011 (2019).

[8] Choi, J. Y., Kim, S., Hwang, B. U., Lee, N. E. & Lee, S. Y. Flexible SiInZnO thin film transistor with organic/inorganic hybrid gate dielectric processed at 150 °C. *Semicond. Sci. Technol.* **31**, 125007 (2016).

[9] Park, J. H, Oh, J. Y, Han, S. W, Lee, T. I. & Baik, H. K. Low-temperature, solution-processed ZrO_2:B thin film: a bifunctional inorganic/organic interfacial glue for flexible thin-film transistors. *ACS Appl. Mater. Interfaces* **7**, 4494-4503 (2015).

[10] Hwang, B.-U. *et al.* Role of ultrathin Al_2O_3 layer in organic/inorganic hybrid gate dielectrics for flexibility improvement of InGaZnO thin film transistors. *Org. Electron.* **15**, 1458-1464 (2014).

[11] Lai, H.-C., Pei, Z., Jian, J.-R. & Tzeng, B.-J. Alumina nanoparticle/polymer nanocomposite dielectric for flexible amorphous indium-gallium-zinc oxide thin film transistors on plastic substrate with superior stability. *Appl. Phys. Lett.* **105**, 033510 (2014).

[12] Rao, M. G. S. *et al.* Tuning the electrical performance of solution-processed In_2O_3TFTs by low-temperature with HfO_2-PVP hybrid dielectric. *Mater. Today Commun.* **26**, 102120 (2021).

[13] Wang, X., Gao, Y., Liu, Z., Luo, J. & Wan, Q. Flexible Low-Voltage IGZO Thin-Film Transistors With Polymer Electret Gate Dielectrics on Paper Substrates. *IEEE Electron Device Lett.* **40**, 224-227 (2019).

[14] Rao, M. G. S., Sánchez-Martinez, A., Gutiérrez-Heredia, G., Quevedo- López, M.A. & Ramírez-Bon, R. Sol-gel derived low temperature HfO_2-GPTMS hybrid gate dielectric for a-IGZO thin-film transistors (TFTs). *Ceram. Int.* **44**, 16428-16434 (2018).

[15] Lin, Y.-H. *et al.* Hybrid organic–metal oxide multilayer channel transistors with high operational stability. *Nat. Electron.* **2**, 587-595 (2019).

[16] Abdullah, I. *et al.* Bias stability of solution-processed In_2O_3 thin film transistors. *J. Phys. Mater.* **4**, 015003 (2020).

[17] Lee, J.-M., Cho, I.-T., Lee, J.-H. & Kwon, H.-I. Bias-stress-induced stretched-exponential time dependence of threshold voltage shift in InGaZnO thin film transistors. *Appl. Phys. Lett.* **93**, 093504 (2008).

[18] Khim, D., Lin, Y. H. & Anthopoulos, T. D. Impact of Layer Configuration and Doping on Electron Transport and Bias Stability in Heterojunction and Superlattice Metal Oxide Transistors. *Adv. Funct. Mater.* **29**, 1902591 (2019).

[19] Chowdhury, M. D. H., Ryu, S. H., Migliorato, P. & Jang, J. Effect of annealing time on bias stress and light-induced instabilities in amorphous indium–gallium–zinc-oxide thin-film transistors. *J. Appl. Phys.* **110**, 114503 (2011).

[20] Kim, Y. H. *et al.* Flexible metal-oxide devices made by room-temperature photochemical activation of sol-gel films. *Nature* **489**, 128-132 (2012).

[21] Park, J. H. *et al.* Low-temperature, high-performance solution-processed thin-film transistors with peroxo-zirconium oxide dielectric. *ACS Appl. Mater. Interfaces* **5**, 410-417 (2013).

[22] Li, J., Huang, C.-X. & Zhang, J.-H. Tuning the electrical performance and bias stability of a semiconducting SWCNT thin film transistor with an atomic layer deposited $AlZrO_x$ composite. *RSC Adv.* **7**, 52517-52523 (2017).

[23] Kim, Y. G., Avis, C. & Jang, J. Low Voltage Driven, Stable Solution-Processed Zinc-Tin-Oxide TFT with HfO_y and AlO_x Stack Gate Dielectric. *ECS Solid State Lett.* **1**, Q23-Q25 (2012).

[24] Yang, J., Yang, X., Zhang, Y., Che, B., Ding, X. & Zhang, J. Improved gate bias stressing stability of IGZO thin film transistors using high-k compounded ZrO_2/HfO_2 nanolaminate as gate dielectric. *Mol. Cryst. Liq. Cryst.* **676**, 65-71 (2019).
